# Flowerbed-inspired biomimetic 3D-printed scaffolds functionalized with urine-derived stem cell exosomes promote alveolar bone regeneration by regulating energy metabolism

**DOI:** 10.7150/thno.123700

**Published:** 2026-01-14

**Authors:** Yanxi Chen, Xiuyuan Yang, Yuxin Zhang, Min Yang, Hongwei Dai, Jie Li, Jianping Zhou

**Affiliations:** 1 The Affiliated Stomatological Hospital of Chongqing Medical University, Chongqing, China.; 2 Chongqing Key Laboratory of Oral Diseases and Biomedical Sciences, Chongqing, China.; 3 Chongqing Municipal Key Laboratory of Oral Biomedical Engineering of Higher Education, Chongqing, China.; 4 Chongqing Municipal Health Commission Key Laboratory of Oral Biomedical Engineering, Chongqing, China.

**Keywords:** 3D printing, bone regeneration, decellularized extracellular matrix, exosome, energy metabolism

## Abstract

**Rationale:** The anatomical complexity and restricted regenerative potential of alveolar bone defects create a significant clinical challenge and highlight the need for spatially biomimetic and biologically supportive biomaterials.

**Methods:** We developed a bone-mimicking matrix hydrogel scaffold inspired by the features of a “flowerbed,” utilizing machine learning-guided three-dimensional bioprinting. Gelatin methacrylate (GelMA), decellularized bone matrix (DBM), and urine-derived stem cell exosomes (USC-Exos) were co-integrated during the printing process to deliver crucial biophysical and biochemical signals for bone regeneration.

**Results:** The GelMA/DBM/USC-Exos scaffold exhibited high printing fidelity, enabling precise fabrication of defect-specific geometries while preserving exosome bioactivity and achieving sustained release (> 16 days). Functionally, the scaffold promoted M2 macrophage polarization and markedly upregulated osteogenic and angiogenic gene expression, which was approximately 2-fold higher than that of the control (p < 0.01). Mechanistically, the scaffold enhanced oxidative phosphorylation by activating the AMP-activated protein kinase pathway, resulting in a nearly 2-fold increase in adenosine triphosphate content and promoting the osteogenic differentiation of jawbone marrow-derived mesenchymal stem cells. *In vivo* implantation in mandibular defect models induced robust neovascularization and bone formation, resulting in a nearly 3-fold increase in vessel density and 65.6 ± 3.0% new bone volume after 4 and 8 weeks, respectively, effectively promoting coordinated and functional alveolar bone regeneration.

**Conclusions:** This study establishes a biomimetic approach that integrates structural biomimicry, exosome-mediated bioactivity, and energy metabolism regulation, offering a promising and targeted strategy for personalized alveolar bone regeneration.

## Introduction

Alveolar bone defects, commonly resulting from trauma, periodontitis, or congenital anomalies, present significant clinical challenges in oral and maxillofacial surgery, as they can impair mastication, phonation, and facial esthetics, ultimately reducing quality of life 1. Although recent advances in bone graft design are remarkable, their clinical translation remains limited due to their inability to accurately recapitulate the complex, patient-specific spatial and structural characteristics of the native osteogenic microenvironment 2. Moreover, bone regeneration is a highly coordinated process involving the synchronized formation of bone and vascular networks, as well as the modulation of dynamic immune responses 3. Consequently, bioactive, spatially biomimetic, and anatomically matched scaffolds that can reconstruct a native-like osteogenic microenvironment and guide interrelated processes are urgently needed.

Bioactive scaffolds that mimic the extracellular matrix (ECM) provide a favorable microenvironment for cell adhesion, migration, and lineage-specific differentiation. Among ECM-mimetic materials, decellularized extracellular matrix (dECM) materials, particularly decellularized bone matrix (DBM), have garnered considerable attention due to their ability to preserve the native ultrastructure, biophysical integrity, and biochemical composition essential for bone regeneration. Recent studies have focused on developing DBM-based materials, from particulate structures to hydrogel composites, to mimic the natural composition of bone tissue 4-7. However, the regenerative performance of conventional DBM hydrogels is limited because they cannot reproduce the anatomical complexity and hierarchical organization of native bone, resulting in suboptimal spatial guidance and tissue integration. To overcome these limitations, three-dimensional (3D) printing has emerged as a promising strategy to fabricate scaffolds with precise geometries and customizable microarchitectures 8. Notably, dECM-based bioinks have demonstrated significant potential in regenerating complex tissues, including cardiac, skin, and renal structures 9. Building on these advances, integrating the biochemical cues of DBM with spatially defined 3D-printed architectures represents a promising approach for functional alveolar bone regeneration.

While 3D-printed DBM scaffolds can replicate structural features, the decellularization process inevitably removes essential bioactive cues, compromising their ability to orchestrate complete bone regeneration. Embedding exosomes in dECM-based scaffolds can compensate for this loss, considerably enhancing scaffold functionality and regenerative outcomes 5,10,11. Exosomes can deliver various bioactive molecules to modulate cellular behavior, facilitate intercellular communication, and reduce the risk of immune rejection. Furthermore, exosomes can directly enhance mitochondrial function by delivering mitochondrial components or indirectly influence metabolic states by activating energy-related signaling pathways 12-14. Exosome integration with various dECM-derived bioinks can effectively promote tissue-specific regeneration. For instance, adipose-derived stem cell exosomes were incorporated into decellularized bone and cartilage matrix bioinks, markedly enhancing cartilage and subchondral bone regeneration 5. Furthermore, tendon-derived stem cell exosomes embedded in injectable tendon hydrogels restored the native tendon microenvironment and improved repair efficiency 10. In contrast, cartilage dECM hydrogels combined with bone marrow mesenchymal stem cell exosomes alleviated osteoarthritis 15. Among the various sources of exosomes, urine-derived stem cells (USCs) have garnered increasing attention due to their non-invasive accessibility, high yield, and regenerative potential 16. Exosomes derived from USCs (USC-Exos) exhibit immunomodulatory capabilities and the ability to induce robust osteogenic differentiation and neovascularization, highlighting their potential as promising candidates for bone tissue engineering applications 17-19. However, whether USC-Exos and dECM scaffolds can exert superior synergistic regenerative effects, and the mechanisms underlying their interactions, are not yet fully understood. Clarifying this interplay is crucial for advancing the therapeutic potential of dECM-exosome hybrid systems and elucidating how exosome-matrix coupling drives coordinated osteogenic regeneration.

**Scheme 1:**
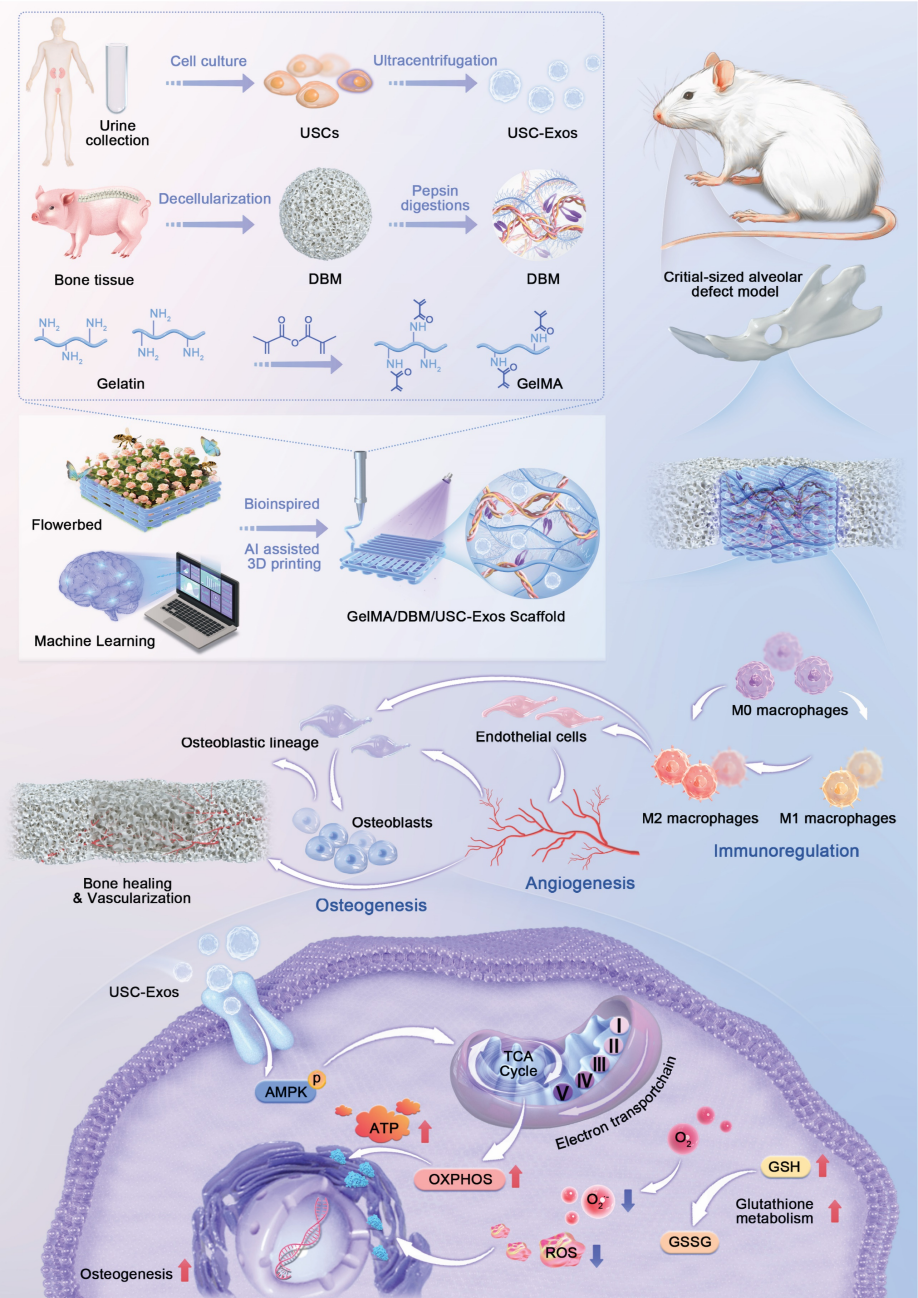
Schematic depicting the fabrication process, therapeutic application, and proposed mechanism of GelMA/DBM/USC-Exos scaffolds that promote alveolar bone defect repair.

To bridge this gap, we aimed to construct a multifunctional scaffold that integrates structural biomimicry with exosome-mediated bioactivity. Inspired by the structural and biological characteristics of “flowerbeds”, we developed a 3D-printed bone-mimicking scaffold composed of gelatin methacrylate (GelMA) and DBM hydrogel, functionalized with USC-Exos, to treat alveolar bone defects (Scheme 1). In this design, GelMA/DBM serves as the structural framework, providing both mechanical support and a cell-friendly microenvironment. At the same time, USC-Exos act as signaling molecules and regenerative cells function as responders, thus reconstructing a biomimetic osteogenic microenvironment. We hypothesized that incorporating USC-Exos into the GelMA/DBM matrix would enhance coordinated bone regeneration by integrating biophysical support with biochemical signaling. To optimize scaffold formulation and ensure high printing fidelity, we employed a machine learning approach to determine the optimal DBM concentration for 3D printing. Comprehensive evaluation of the scaffold's physicochemical properties, biological functions, and regenerative efficacy was conducted *in vitro* and *in vivo*. We also investigated the mechanisms underlying the USC-Exos-based delivery system, focusing on its capacity to regulate osteogenic differentiation by modulating cellular energy metabolism. Our strategy of integrating extracellular matrix-mimicking architecture and exosome-mediated bioactivity into a biocompatible scaffold presents a promising approach for promoting alveolar bone regeneration.

## Methods

### Cell isolation and culture

Primary USCs were isolated from urine samples provided by healthy male donors aged 20-30 years. Jawbone marrow-derived mesenchymal stem cells (JBMSCs) were isolated from bone fragments collected during orthognathic surgery from age-matched donors following established protocols 16,20. Mesenchymal stem cell (MSC) characteristics were confirmed using osteogenic and adipogenic differentiation assays and flow cytometry. Detailed procedures are described in the Supplementary Material.

### Isolation and characterization of USC-derived exosomes

Once USCs reached approximately 60%-70% confluence, they were transferred to culture medium supplemented with 10% exosome-depleted fetal bovine serum, which had been pre-cleared by ultracentrifugation at 100,000 × g for 16 h. After 24-48 h of incubation, the conditioned medium (CM) was collected and subjected to stepwise centrifugation and ultracentrifugation (100,000 × g, 2 h, 4 °C, Beckman Coulter, USA) for exosome isolation. Exosome characterization included nanoparticle tracking analysis, transmission electron microscopy (TEM), and western blotting. The osteogenic induction potential of USC-Exos on JBMSCs was evaluated using cell counting kit-8 (CCK-8), alkaline phosphatase (ALP) staining, and real-time quantitative polymerase chain reaction (RT-qPCR). Detailed procedures and antibody information are provided in the Supplementary Material.

### Preparation and characterization of DBM-based bioink

DBM was obtained from porcine vertebral trabecular bone using a modified protocol for bioink preparation based on previous reports 5,21. Bone fragments were cut into small cubes, rinsed in sterile phosphate-buffered saline (PBS) for 24 h, and sequentially treated under agitation (300 rpm) for decellularization, as follows: 0.5 N HCl (24 h) for demineralization, 1% Triton X-100 (12 h) for cell lysis, methanol (6 h) for lipid removal, and 100% ethanol (4 h) for DNA elimination, with interspersed PBS washes. The resulting DBM was lyophilized and stored at -20 °C for further use.

The DBM and native bone samples were fixed (4% paraformaldehyde), dehydrated, embedded, and sectioned. Hematoxylin and eosin (H&E) staining (Solarbio, China) was used to assess residual cells, picrosirius red (Solarbio, China) was used to evaluate collagen distribution, alcian blue (Solarbio, China) was used to detect sulfated glycosaminoglycan (sGAG), and 4',6-diamidino-2-phenylindole (DAPI) was used for nuclear visualization. Quantitative assays included double-stranded DNA content (TIANGEN, China), sGAG quantification (Chondrexr, USA), and collagen measurement (Abcam, USA) according to the manufacturer's protocols.

Freeze-dried DBM was milled into a fine powder and enzymatically digested in hydrochloric acid containing pepsin (0.1 M; 10 mg per 100 mg DBM) for 3 days. After digestion, the suspension was centrifuged at 4,000 rpm for 10 min to remove undissolved particulates. The pH of the resulting supernatant was adjusted to approximately 7.4 using pre-chilled NaOH (10 M). The neutralized DBM solution was stored at 4 °C until further use. GelMA solutions were obtained by dissolving lyophilized GelMA and the photoinitiator lithium phenyl-2,4,6-trimethylbenzoylphosphinate (SunP, China) in PBS. Composite bioinks were then formulated by mixing GelMA with varying concentrations of DBM to yield the following formulations: 1) 10% (w/v) GelMA + 1% DBM; 2) 10% GelMA + 2% DBM; 3) 10% GelMA + 3% DBM; and 4) 10% GelMA + 4% DBM. A 10% (w/v) GelMA solution without DBM served as the control.

The rheological characteristics of the GelMA/DBM bioinks were evaluated using a C35 1°/Ti cone rotor (1 mm gap) with a rotational rheometer (Thermo Scientific, USA). Shear viscosity was measured using a shear rate sweep (0.1-100 s⁻¹) at 25 °C. Storage (G') and loss (G”) moduli were determined using a frequency sweep (0.1-10 Hz) under a strain of 0.01 at 25 °C. Temperature-dependent behavior was assessed using a temperature sweep from 10 °C to 40 °C (1 Hz frequency). Swelling and degradation analyses were performed as detailed in the Supplementary Material.

To assess the osteoinductive capacity of DBM-based bioinks, cylindrical constructs of equal volume were fabricated from each formulation and photo-crosslinked under 405 nm ultraviolet (UV) light for 30 s. JBMSCs (1 × 10⁶ cells/well) were co-cultured with the constructs using osteogenic induction medium. ALP and Alizarin Red S (ARS) staining were conducted on days 7 and 14, respectively.

### Preparation and characterization of hydrogel scaffolds

#### Machine learning-guided 3D printing optimization

A machine-learning model was developed in MATLAB (The MathWorks, USA) using the Statistics and Machine Learning Toolbox. The model was trained and validated using experimental datasets from hydrogels formulated with 1%, 2%, 3%, and 4% (w/v) DBM, whereas data from the 2.5% group were used for testing. The input variables included DBM concentration, nozzle size, printing temperature, and extrusion speed, and the output was the printability score. An optimizable Gaussian process regression (GPR) model was used to estimate the printability scores of the experimental 3D bioprinting data.

#### 3D printing and printability assessment

Bioinks were loaded into cartridges and preincubated at 25 °C for 10 min before printing. The constructs were fabricated using a 3D bioprinter (SunP, China) equipped with a 23 G nozzle at a printing temperature of 25 °C and a constant platform temperature of 4 °C. Hydrogel samples (10 × 10 × 1 mm) were printed at a predefined extrusion speed. For GelMA/DBM/USC-Exos scaffolds, freshly isolated exosomes were incorporated into the GelMA/DBM bioink at a final concentration of 50 μg/mL. The constructs were photocrosslinked under UV light after printing.

#### Morphological and structural characterization

The microstructures of the printed scaffolds were examined using scanning electron microscopy (SEM; Thermo Scientific, USA). For chemical characterization, freeze-dried hydrogels were ground into a powder and analyzed by Fourier transform infrared (FTIR) spectroscopy (KBr pellet method, 4000-400 cm⁻¹; Thermo Scientific, USA) to confirm the synthesis of GelMA and GelMA/DBM hydrogels. Square hydrogels were prepared for mechanical testing. Compressive strength was measured using a universal testing machine (SANS, China) at a compression rate of 1 mm/min and a maximum strain of 50%. To assess fatigue resistance, scaffolds were subjected to ten consecutive compression cycles at a constant rate, and stress-strain curves were recorded for the 1st, 5th, and 10th cycles. The procedures, swelling behavior, and degradation kinetics are described in detail in the Supplementary Material.

#### Exosome labeling

Exosomes were labeled with PKH26 red fluorescent dye (Solarbio, China) and incorporated into the GelMA/DBM bioink to fabricate GelMA/DBM/USC-Exos scaffolds. Exosome distribution within the hydrogels was visualized using confocal laser scanning microscopy (CLSM; Leica, Germany).

#### Release profile of exosomes

Exosome release kinetics from the hydrogel scaffolds were assessed using a BCA Protein Assay Kit (Beyotime, China). GelMA/DBM and GelMA/DBM/USC-Exos scaffolds were placed in 8 μm Transwell inserts (Corning, USA) in 12-well plates, with PBS added to the lower chambers. At predetermined time points, PBS (15 μL) was collected and replaced with fresh PBS. GelMA/DBM scaffolds without exosomes served as controls to correct for nonspecific protein release. The exosomal protein content in the lower chamber was quantified. For *in vivo* tracking of exosome retention, DiR-labeled USC-Exo-loaded hydrogels were implanted into rat mandibular defects. Fluorescence signals were monitored at predetermined time points using an *in vivo* imaging system (IVIS, Revvity, USA) with consistent exposure settings.

#### Cellular uptake of USC-Exos

For internalization studies, USC-Exos (50 μg/mL) were fluorescently labeled with PKH26 dye (Solarbio, China) and incorporated into the GelMA/DBM pre-gel solution to form composite scaffolds. Labeled scaffolds were co-cultured for 24 h with (1) phorbol 12-myristate 13-acetate (PMA)-induced THP-1-derived macrophage-like cells, (2) human umbilical vein endothelial cells (HUVECs), and (3) JBMSCs. After incubation, the cells were fixed and stained with phalloidin (Solarbio, China) and DAPI (Beyotime, China) to visualize the cytoskeleton and nucleus, respectively. The cellular uptake and intracellular distribution of USC-Exos were analyzed using CLSM (Leica, Germany).

#### Cytotoxicity of hydrogel scaffolds

JBMSCs and HUVECs (2 × 10⁴ cells/well) were co-cultured with various hydrogel scaffolds using a Transwell system (Corning, USA). After 3 days, cell viability was examined using a Calcein-AM/Propidium Iodide dual-staining kit (Elabscience, China). Cell proliferation was assessed using a CCK-8 assay (Beyotime, China) on days 1, 4, and 7, and 5-ethynyl-2'-deoxyuridine (EdU) incorporation (Beyotime, China) was performed on day 3 to assess the proliferative responses to different hydrogel formulations.

#### Cell adhesion and morphology on hydrogel scaffolds

JBMSCs and HUVECs were co-cultured on each hydrogel scaffold for one day. After fixation, the actin filaments and nuclei were stained with phalloidin (Solarbio, China) and DAPI (Beyotime, China), respectively. Cytoskeletal organization and cell morphology were visualized using CLSM (Leica, Germany), and cell attachment and spreading on the scaffold surfaces were further examined using SEM.

### *In vitro* immunomodulatory evaluation

THP-1 monocytes were differentiated into macrophages by treatment with 100 ng/mL PMA (MCE, China) for 24-48 h until cell adherence was observed. Differentiated macrophages (5 × 10⁵ cells/well) were seeded and co-cultured with hydrogel scaffolds using a Transwell system (Corning, USA). To induce an inflammatory response, 1 mg/mL lipopolysaccharide (LPS; Sigma, USA) was added, followed by a 24 h incubation period. The immunomodulatory effects of the scaffolds were assessed using immunofluorescence staining, qPCR, ELISA, and western blotting. Detailed protocols are provided in the Experimental Section of the Supplementary Material.

### *In vitro* angiogenic evaluation

The angiogenic potential of the hydrogels was assessed using immunofluorescence staining, qPCR, ELISA, and tube formation assays. Details of the experimental procedures and analysis methods are provided in the Supplementary Material.

### Transwell migration assay and scratch test

For the Transwell assay, JBMSCs and HUVECs (2 × 10⁵ cells) were seeded into the upper chambers of 8 μm inserts (Corning, USA), with hydrogel scaffolds placed in the lower chambers of a 12-well plate. After 24 h, the migrated cells were fixed (4% paraformaldehyde), stained (0.1% crystal violet), and imaged using optical microscopy.

For the scratch assay, confluent JBMSC and HUVEC monolayers (2 × 10⁵ cells/well) were scratched and co-cultured with hydrogel scaffolds placed in Transwell inserts. Images were captured at 0 h and 24 h using optical microscopy.

### ALP and ARS staining

JBMSCs (1 × 10⁶ cells/well) were co-cultured with different hydrogel formulations using a Transwell system in osteogenic induction medium. For the positive control, recombinant human BMP-2 (100 ng/mL; Abclonal, China) was added to the culture medium. ALP and ARS staining were conducted to assess osteogenic activity.

### RNA-sequencing

JBMSCs were co-cultured with the GelMA/DBM or GelMA/DBM/USC-Exos scaffolds for 7 days, followed by total RNA extraction for RNA sequencing. Complementary DNA library construction and high-throughput sequencing were performed by LC Biotech Co., Ltd. (China). Each group included three independent biological replicates. Differential gene expression analysis was performed using standard bioinformatics pipelines; genes showing a false discovery rate-adjusted q-value < 0.05 were defined as differentially expressed genes (DEGs).

### Seahorse assay

After 7 days of co-culture with the GelMA/DBM and GelMA/DBM/USC-Exos scaffolds, JBMSCs (2 × 10^4^ cells/well) were seeded into XF96 cell culture microplates. Metabolic parameters, including the oxygen consumption rate (OCR) and the extracellular acidification rate (ECAR), were measured using a Seahorse XFe96 Extracellular Flux Analyzer (Agilent Technologies, USA). Further technical details and assay conditions are provided in the Supplementary Material.

### ATP level determination and NADH/ NAD⁺ ratio measurement

After 7 days of co-culture with the GelMA/DBM and GelMA/DBM/USC-Exos scaffolds, the intracellular adenosine triphosphate (ATP) content, and reduced nicotinamide adenine dinucleotide (NADH)/oxidized nicotinamide adenine dinucleotide (NAD⁺) levels were measured using ATP and Enhanced NADH/NAD⁺ Assay Kits (Beyotime, China).

### Mitochondrial morphology analysis

To assess mitochondrial ultrastructure, JBMSCs were fixed in 2.5% glutaraldehyde. After fixation, the cells were dehydrated, embedded in resin, sectioned into ultrathin slices, and examined by TEM.

### Effect of an AMPK inhibitor on mitochondrial respiration and osteogenic differentiation of JBMSCs

Western blot analysis determined 10 μM as the optimal working concentration of the AMP-activated protein kinase (AMPK) inhibitor, Compound C. JBMSCs were then co-cultured for 7 days with GelMA/DBM scaffolds, GelMA/DBM/USC-Exos scaffolds, or GelMA/DBM/USC-Exos scaffolds supplemented with Compound C. Mitochondrial respiration was assessed using a Seahorse XFe96 Analyzer (Agilent Technologies, USA). ALP and ARS staining were performed to evaluate osteogenic differentiation.

### ROS and redox status detection

JBMSCs (2 × 10⁴ cells/well) were co-cultured with GelMA/DBM or GelMA/DBM/USC-Exos scaffolds using a Transwell system. Intracellular reactive oxygen species (ROS) levels were assessed using an ROS Assay Kit (Beyotime, China). Redox homeostasis was assessed by quantifying reduced glutathione (GSH) and oxidized glutathione (GSSG) levels with a GSH/GSSG Assay Kit (Beyotime, China).

### *In vivo* bone repair study

#### Critical-size alveolar bone defect repair assay

A critical-sized alveolar bone defect model was established in male Sprague-Dawley rats to evaluate the *in vivo* immune response, neovascularization, and bone regeneration. All procedures were approved by the Ethics Committee of the College of Stomatology, Chongqing Medical University (CQHS-REC-2024 (LSNo.119)). Standardized circular defects (3 mm in diameter) were created in the mandibles of 6-8-week-old Sprague-Dawley rats (n = 6 per group). Sterilized hydrogel scaffolds (GelMA, GelMA/DBM, or GelMA/DBM/USC-Exos) were implanted into the defect sites. Untreated defects served as controls. Animals were euthanized 4 and 8 weeks after implantation to assess bone regeneration. The mandibles were harvested and fixed in 10% neutral-buffered formalin. For immunological analysis, a separate cohort was sacrificed after 1 week to assess macrophage responses. A geometric variant model was created using triangular (3 × 3 × 1 mm) and parallelogram-shaped (3 × 2 × 1 mm) defects. These were divided into control (no scaffold) and treatment (GelMA/DBM/USC-Exos) groups. To assess systemic biocompatibility, major organs were harvested after 4 and 8 weeks for histological analysis. The detailed procedure for the hemolysis assay is provided in the Supplementary Material.

#### Cytokine quantification by ELISA

Supernatants from mandibular defect sites were collected at 1-week post-surgery and stored at -80 °C until analyzed. Cytokines associated with M1/M2 macrophage polarization were quantified using ELISA (Elabsciences).

#### Micro-computed tomography (micro-CT) analysis and nanoindentation test

Mandibular specimens were scanned using Micro-CT (vivaCT40, SCANCO Medical, Switzerland). Three-dimensional reconstructions were generated, and quantitative parameters were calculated to evaluate new bone formation. Details of the nanoindentation testing procedures are provided in the Supplementary Material.

#### Histological, immunohistochemical, and immunofluorescence analysis

Mandibular bone samples were collected 1-, 4-, and 8-weeks post-implantation. H&E, Masson's trichrome, and Goldner's trichrome staining were conducted using commercially available kits (Solarbio, China).

To investigate the biological functions of implanted hydrogels, immunohistochemical staining was performed for interleukin (IL)-1β (inflammation), CD31 (angiogenesis), and ALP and osteopontin (OPN) (osteogenesis). Immunofluorescence staining was used to assess cellular and molecular responses. Markers included CD90 (stem cell recruitment); F4/80, inducible nitric oxide synthase (iNOS), and CD206 (macrophage polarization); vascular endothelial growth factor A (VEGFA; neovascularization); collagen type I (COL-1), and osteocalcin (OCN; bone matrix formation); as well as citrate synthase (CS), isocitrate dehydrogenase 1 (IDH1), and oxidative phosphorylation (OXPHOS)-related proteins (tricarboxylic acid (TCA) cycle and OXPHOS activity). Antibody details are provided in the Supplementary Material.

### Statistical analysis

All experiments were conducted in triplicate, and data are presented as mean ± standard deviation (SD, n = 3). Statistical analyses were performed using SPSS and Origin software. Student's t-test was used to compare two groups, and one-way analysis of variance (ANOVA) was used to compare multiple groups, after normality testing. Statistical significance was defined as P < 0.05, P < 0.01, and P < 0.001.

## Results and discussion

### Preparation and characterization of USCs/USC-Exos and DBM-based bioink

**Figure 1:**
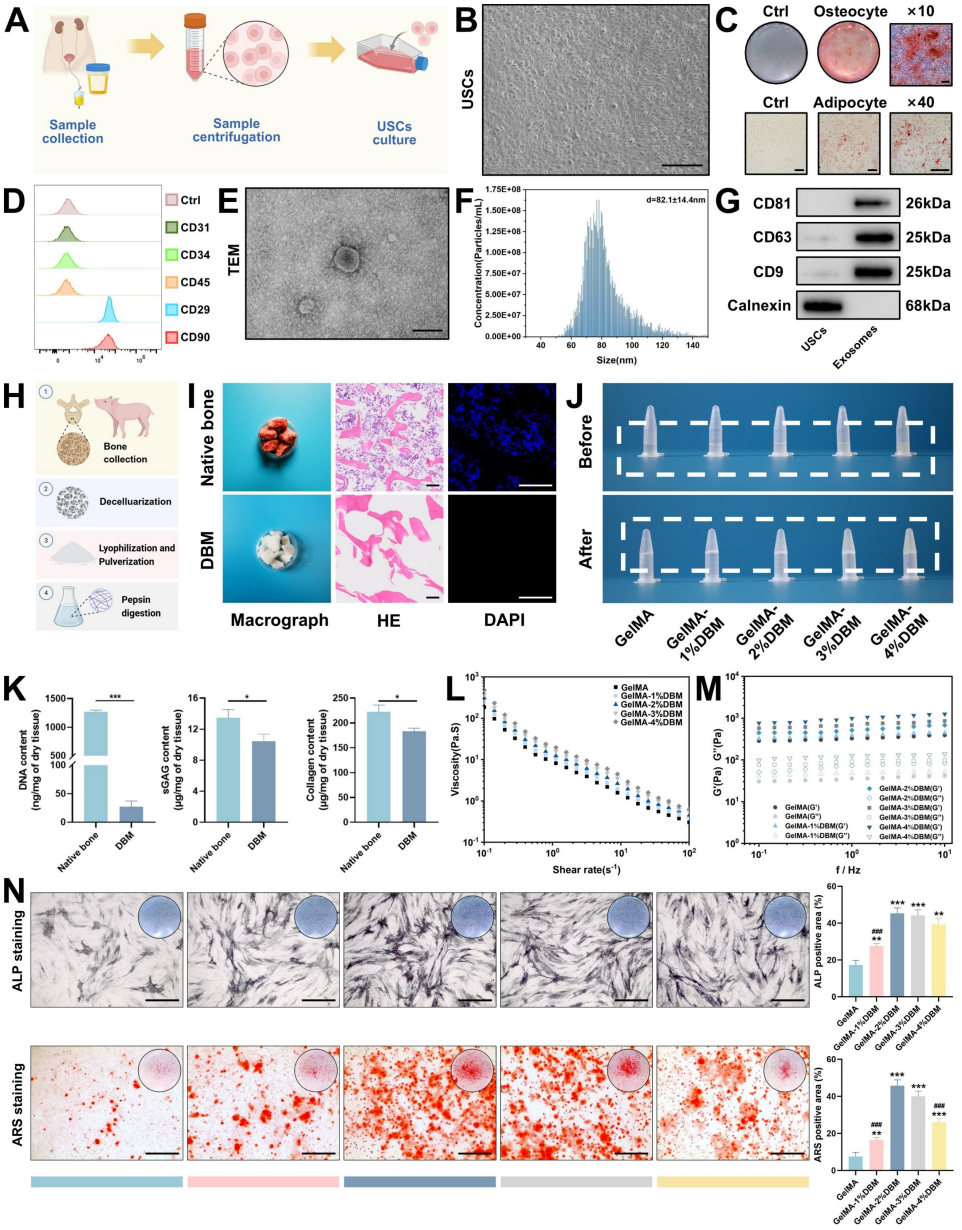
** Characterization of USCs, USC-derived exosomes, and preparation of GelMA/DBM bioink.** (A) Schematic of USC isolation and culture. (B) Optical microscopy image of USCs. Scale bar = 100 μm. (C) Alizarin Red and Oil Red O staining confirmed osteogenic and adipogenic differentiation. Scale bar = 100 μm. (D) Flow cytometric analysis of MSC surface markers. (E) TEM image of USC-Exos. Scale bar = 100 nm. (F) Nanoparticle tracking analysis of USC-Exos. (G) Western blot analysis of exosomal and negative markers in USCs and USC-Exos. (H) Schematic of DBM preparation from porcine bone. (I) Macroscopic and histological (H&E, DAPI) comparison before and after decellularization. Scale bar = 200 μm. (J) Macroscopic appearance of bioinks with varying DBM contents before and after photocrosslinking. (K) Quantification of DNA, sGAG, and collagen in DBM compared with that in native bone. (L-M) Rheological assessment of GelMA/DBM composites under varying shear rates and frequencies. (N) ALP and ARS staining with quantification of osteogenesis in JBMSCs within GelMA/DBM hydrogels at different DBM concentrations. Scale bar = 400 μm. Data are presented as mean ± SD (n ≥ 3). ^*^ P < 0.05, ^**^ P < 0.01, and ^***^ P < 0.001 versus the GelMA group; and ^#^ P < 0.05, ^##^ P < 0.01, and ^###^ P < 0.001 versus the GelMA-2% DBM group.

JBMSCs were selected as target cells to evaluate the regenerative potential of USC-Exos, owing to their physiological relevance in maxillofacial bone repair and the site-specific characteristics of the alveolar bone 22. USCs were collected by centrifuging non-invasive urine samples, whereas JBMSCs were isolated from jawbone tissue (Figures 1A and S1A). Both cell types exhibited typical spindle-shaped, fibroblast-like morphologies under light microscopy (Figures 1B and S1B) and expressed typical mesenchymal surface markers (CD29⁺, CD90⁺, CD31⁻, CD34⁻, and CD45⁻) as confirmed by flow cytometry (Figures 1D and S1C). Stemness was validated by successful osteogenic and adipogenic differentiation (Figures 1C and S1D). CCK-8 proliferation assays showed sustained growth for both cell types over six days, with USCs displaying a slightly higher proliferation rate during the early culture stages, indicating their scalability for exosome production (Figure S2). USC-Exos were isolated from the CMs using differential ultracentrifugation (Figure S3A). They exhibited characteristic cup-shaped morphologies, with an average diameter of 82.1 ± 14.4 nm, expressed classical exosomal markers (CD63, CD81, and CD9), and lacked the intracellular protein calnexin (Figure 1E-G). These results confirmed the successful isolation of both USCs and their derived exosomes.

To evaluate the biological effects of USC-Exos, JBMSCs were co-cultured with varying concentrations of USC-Exos. Proliferation assays (Figure S3B) demonstrated a significant dose- and time-dependent increase in cell growth, with peak proliferation at 50 and 70 μg/mL. ALP staining and quantitative analysis revealed a dose-dependent increase in early osteogenic activity, with the 50 μg/mL group showing the highest ALP-positive area (Figure S3C). Analysis of osteogenic gene expression further supported this observation (Figure S3D). Although the 70 μg/mL group also showed enhanced osteogenic marker expression, the levels were slightly reduced compared with those in the 50 μg/mL group, possibly because of receptor saturation or negative feedback regulation at higher concentrations. Considering the observed biological effects and practical considerations related to exosome production costs, 50 μg/mL was selected as the optimal dose for subsequent investigations.

DBM was prepared from porcine cancellous bone using decellularization and enzymatic digestion, based on modified protocols 5,21 (Figure 1H). After treatment, the red marrow was visibly removed, and the resulting DBM showed an elastic texture (Figure 1I). Histological analyses confirmed complete decellularization; native bone retained cellular components, whereas DBM lacked nuclei and cellular debris, as shown by DAPI staining. The DNA content in DBM was reduced to below 50 ng/mg dry weight, meeting the immunogenicity threshold, and further supporting our findings 21 (Figure 1K). Importantly, the key matrix components, sGAG and collagen, were largely preserved (Figures 1K and S4A), indicating the retention of essential bioactivity. Subsequent pepsin digestion solubilized the collagen aggregates into monomers, forming a translucent, homogeneous DBM solution. Upon warming to 37 °C, the solution underwent a thermosensitive sol-gel transition, resulting in the formation of a hydrogel, as confirmed by rheological analysis (Figure S4B-C).

Native DBM hydrogels exhibit poor printability and limited mechanical strength, which compromise their structural integrity and shape fidelity 4,23. To address these limitations, GelMA was added to improve printability and mechanical stability, as well as to partially restore collagen loss during decellularization. The bioink composition must be optimized to ensure optimal print performance and the creation of a biomimetic microenvironment conducive to cell proliferation and differentiation in 3D printing. Accordingly, DBM was incorporated at 1%, 2%, 3%, and 4% (w/v) into a 10% GelMA solution to generate five composite bioinks. Increasing the DBM content reduced the optical transparency in the liquid state. After photo-crosslinking, samples with a high level of DBM appeared opaque and milky white, whereas the pure GelMA hydrogels remained clear (Figure 1J). Despite these optical changes, the composite hydrogels maintained excellent injectability and moldability (Figure S4D).

Rheological analysis, critical for evaluating the mechanical and printability properties of extrusion-based bioinks, revealed that viscosity (Figure 1L) and dynamic modulus (Figure 1M) increased with the DBM concentration. All formulations exhibited shear-thinning behavior, as evidenced by a linear decrease in viscosity with increasing shear rate, indicating that the incorporation of DBM did not compromise the flow properties of GelMA. The storage modulus (G') exceeded the loss modulus (G”) across all frequencies, indicating predominantly elastic behavior and a stable hydrogel network with mechanical integrity that is suitable for high print fidelity 24. The addition of DBM slightly reduced the swelling ratio compared with that of pure GelMA, suggesting improved structural stability and water retention (Figure S5A). Degradation studies in a collagenase-rich simulated bone environment (1 µg/mL of collagenase I and II) revealed that > 95% of pure GelMA degraded within 16 days. In contrast, DBM-containing hydrogels degraded more slowly (Figure S5B). This enhanced enzymatic resistance was likely due to the dense, collagen-rich matrix of the DBM and potential intermolecular interactions with GelMA, which contributed to a more stable hydrogel network.

To determine the optimal osteoinductive formulation, JBMSCs were cultured in each hydrogel variant under osteogenic conditions. ALP and ARS staining revealed that DBM incorporation significantly enhanced the osteoinductive capacity of GelMA hydrogels, with the 2% and 3% DBM groups exhibiting the highest ALP activity and calcium deposition (Figure 1N). In contrast, the 4% DBM group exhibited a slight reduction in osteogenic markers, likely due to impaired nutrient diffusion or disrupted cell-matrix interactions resulting from the excessive density of the matrix. Overall, the DBM-incorporated composite hydrogels exhibited osteoinductive and osteoconductive characteristics, consistent with previous studies 4,6.

### Fabrication and physicochemical properties of 3D-printed hydrogel scaffolds

#### Machine learning-assisted optimization of 3D printing parameters

“Flowerbeds” are deliberately designed by horticulturists, typically adorned with a variety of plants and flowers, and enclosed by functional fences to define boundaries and offer protection. When in bloom, the flowers release pollen and attract beneficial insects such as bees and butterflies, creating a dynamic microecosystem. Inspired by the structural features of a flowerbed, we engineered a biomimetic scaffold integrated with bioactive cues using 3D printing techniques. In recent years, 3D printing with diverse biomaterials has gained traction in tissue engineering because of its advantages in constructing complex architectures, customizing patient-specific implants, and improving time and cost efficiencies 8,9. Despite the progress in hydrogel-based 3D printing, optimizing the parameters for new formulations remains a key challenge. To address this, we developed a GelMA/DBM-based hydrogel ink and optimized its printability using a machine learning-assisted model. Scaffolds with mesh structures (10 × 10 × 1 mm) were fabricated using a pneumatic bioprinter. Key variables, including the nozzle gauge (23 G and 27 G), extrusion speed (1 and 2 mm/s), and DBM concentration (1%, 2%, 2.5%, 3%, and 4% w/v), were adjusted independently while maintaining a fixed nozzle movement speed of 3 mm/s. Printing was performed at 25 ℃ and 30 ℃ during the GelMA/DBM sol-gel transition, ensuring optimal printability by maintaining a semi-solid state that balanced flow and shape fidelity (Figure S5C). Printability was assessed using a structured scoring system adapted from a previous method 25. The following criteria were evaluated: (i) filament continuity (scored as 2 for continuous, 1 for partial, and 0 for failed strands), (ii) dimensional fidelity in both the horizontal and vertical planes (scored as 4, 2, or 0 based on conformity to the designed geometry), and (iii) vertical accuracy in height (scored as 6 for excellent, 4 for good, 2 for fair, and 0 for poor). The total scores were summed to enable quantitative comparisons across different parameter combinations.

**Figure 2:**
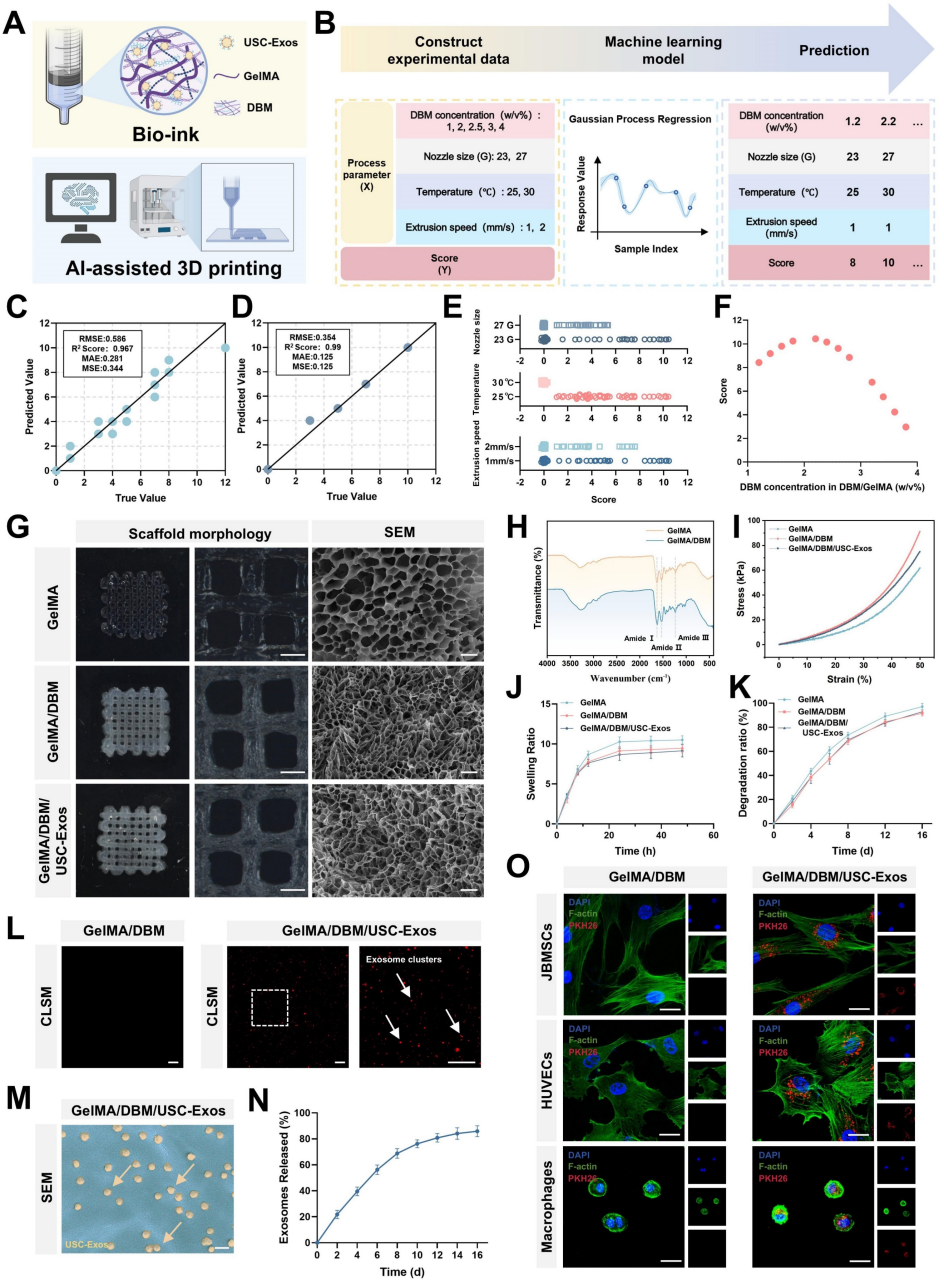
** AI-assisted optimization, fabrication, and characterization of GelMA/DBM/USC-Exos hydrogel scaffolds.** (A) Schematic of bioink formulation using GelMA, DBM, and USC-Exos for AI-guided 3D bioprinting. (B) Workflow of printability optimization via GPR. (C-D) Validation and testing of the trained GPR model. (E-F) Printability scores under varying nozzle sizes, temperatures, extrusion speeds, and DBM concentrations. (G) Macroscopic (scale bar = 500 μm) and SEM (scale bar = 20 μm) images of printed scaffolds. (H) FTIR spectra of GelMA and GelMA/DBM hydrogels. (I-K) Compressive stress-strain behavior, swelling capacity, and degradation profiles. (L) CLSM-based visualization of PKH26-labeled exosome distribution within the GelMA/DBM and GelMA/DBM/USC-Exos scaffolds. Scale bar = 20 μm. (M) SEM image of the GelMA/DBM/USC-Exos scaffold. Scale bar = 500 nm. (N) Cumulative exosome release profile. (O) Cellular uptake of PKH26-labeled exosomes by JBMSCs, HUVECs, and THP-1 macrophages. Scale bar = 20 μm. Data are presented as mean ± SD (n ≥ 3).

A schematic of the machine learning-based framework for predicting hydrogel printability is shown in Figure 2A-B. A GPR algorithm with a rational quadratic kernel was selected due to its robustness with small datasets and ability to quantify predictive uncertainty, and was used to construct a predictive model 26. Owing to its flexibility and predictive accuracy, GPR has demonstrated strong performance in various engineering and biological domains 26-29. All predicted values fell within the 95% confidence interval with narrow bounds, suggesting a high degree of precision. (Figure S6). The model was trained using DBM concentrations of 1%, 2%, 3%, and 4% (w/v), and its predictive accuracy was validated using a testing set with a DBM concentration of 2.5% (w/v). The model demonstrated excellent performance as the validating and testing datasets yielded R² values of 96.7% and 99.0%, respectively (Figure 2C-D), indicating a robust fit and generalizability. To refine the optimal DBM concentration, the trained model simulated 96 combinations across finer increments (1.2%-3.8% DBM, in 0.2% steps) under varied print settings. The analysis revealed that a larger nozzle size (23 G), lower temperature (25 °C), and slower extrusion speed (1 mm/s) were associated with higher printability scores (Figure 2E). Under optimal conditions, peak scores were observed at DBM concentrations of approximately 1.8% and 2.2% (w/v), with a decline at higher concentrations, likely due to excessive viscosity that impaired extrusion fidelity (Figure 2F). Considering both model predictions and prior biological data, 2% DBM was selected as the optimal concentration to balance print precision and osteoinductive efficacy for subsequent scaffold fabrication.

#### Structural characterization and physicochemical evaluation of GelMA/DBM/USC-exos scaffolds

A 3D printing test was conducted using scaffold constructs (10 × 10 × 1 mm) under optimized conditions. Compared with freeze-dried hydrogels, which absorb water and randomly adsorb exosomes in a non-uniform manner, 3D printing enables a more homogeneous distribution of exosomes within the scaffold matrix 5. Thus, USC-Exos were incurporated into the GelMA/DBM pregel, and composite scaffolds (GelMA/DBM/USC-Exos) were fabricated via photocrosslinking. All of the scaffolds displayed well-ordered lattice structures with a hierarchical microstructure and pore sizes ranging from 400 to 600 µm, which are optimal characteristics for promoting angiogenesis and osteogenesis (Figure 2G) 30. All freeze-dried scaffolds observed using SEM exhibited interconnected microporous networks, essential for efficient oxygen diffusion and nutrient transport. Quantitative analysis revealed that GelMA scaffolds had an average pore size of 16.2 ± 4.7 µm. In contrast, GelMA/DBM and GelMA/DBM/USC-Exos scaffolds showed reduced pore sizes of 9.2 ± 3.2 µm and 9.8 ± 3.3 µm, respectively (Figure S7A). These results are attributed to the densifying effect of DBM incorporation. Notably, exosome loading did not significantly affect porosity, indicating that the scaffold microarchitecture was preserved.

In addition to morphological analysis, FTIR was performed to assess the functional groups and molecular interactions within the crosslinked GelMA/DBM hydrogel scaffolds. FTIR spectra confirmed successful DBM incorporation, showing distinct amide I (~1627 cm⁻¹), amide II (~1535 cm⁻¹), and amide III (~1235 cm⁻¹) peaks, corresponding to C=O stretching, N-H bending, and C-N stretching, respectively (Figure 2H). These signals reflected the presence of collagen-rich components derived from DBM.

Effective bone regeneration requires biomaterials with sufficient mechanical strength to support physiological loads and guide tissue remodeling. Compressive testing was performed to evaluate the mechanics of the scaffold (Figures 2I and S7B). Stress-strain curves revealed that pure GelMA hydrogels exhibited the lowest compressive modulus (36.89 ± 3.09 kPa), indicating limited structural support. DBM incorporation significantly enhanced stiffness, with GelMA/DBM and GelMA/DBM/USC-Exos scaffolds reaching 55.01 ± 3.53 kPa and 52.99 ± 3.00 kPa, respectively.

Given that elastin and sGAG are key ECM components known to strengthen bone mechanical properties 4, the improvement was attributed to the reinforcement of the hydrogel network by the collagen-rich DBM matrix. Notably, under cyclic compression, the pure GelMA scaffold exhibited progressive deformation after 5 and 10 loading cycles, whereas the incorporation of DBM effectively maintained structural stability, resulting in stress-strain curves that remained almost unchanged (Figure S7C). Thus, the composite scaffolds exhibited superior fatigue resistance and mechanical stability, providing favorable mechanical cues for bone-organoid formation and functional integration.

Scaffolds with adequate water absorption are crucial for facilitating nutrient exchange and waste removal within the cellular microenvironment. All groups exhibited rapid initial swelling within the first 24 h, likely due to the high porosity and flexibility of the freeze-dried structures, which facilitated water diffusion (Figure 2J). The GelMA/DBM and GelMA/DBM/USC-Exos scaffolds showed slightly lower swelling ratios (approximately 8.7 and 9.1, respectively), which may have resulted from their smaller pore sizes. This reduced porosity enhanced dimensional stability and reduced deformation under physiological conditions.

To assess *in vitro* degradation, freeze-dried scaffolds were incubated in PBS containing collagenase I and II (1 µg/mL each), and the residual mass was measured over time. Both the GelMA/DBM and GelMA/DBM/USC-Exos scaffolds degraded more slowly than pure GelMA, with final degradation ratios of approximately 91.6% and 92.8%, respectively, compared with approximately 97.1% for GelMA by day 16 (Figure 2K). The slower degradation is attributed to DBM reinforcement, which enhances matrix stability and enzymatic resistance. These results highlight the favorable biodegradation profile of the GelMA/DBM/USC-Exos scaffold, supporting its suitability for bone regeneration, which demands prolonged structural support and gradual resorption.

To assess exosome encapsulation and release, PKH26-labeled USC-Exos were incorporated into the bioink and printed onto the scaffold structures. CLSM confirmed successful encapsulation and uniform distribution within the hydrogel matrix (Figure 2L). These results were supported by SEM imaging, which showed exosome-like structures embedded in the scaffold (Figure 2M). Release profiling revealed a sustained release pattern, with cumulative exosome release reaching approximately 56.2% by day 6 and exceeding 85% by day 16 (Figure 2N), closely aligning with the scaffold degradation kinetics. The IVIS showed localized fluorescence at the implantation site up to day 15, with gradually decreasing intensity over time (Figure S8), indicating sustained retention and controlled release of exosomes *in vivo*. This prolonged release is favorable for maintaining a therapeutic window conducive to continuous tissue remodeling and regeneration, highlighting the potential of GelMA/DBM hydrogels as effective carriers for bioactive delivery in regenerative applications. To further validate the functional activity of embedded exosomes, PKH26-labeled USC-Exo-loaded scaffolds were co-cultured with three cell types, JBMSCs, HUVECs, and THP-1 macrophages, all of which play crucial roles in alveolar bone regeneration. CLSM confirmed efficient uptake by all cell types (Figure 2O), indicating preserved functionality post-encapsulation and supporting their roles in osteogenesis, angiogenesis, and immune modulation during bone healing.

### Evaluation of cytocompatibility and cell adhesion on GelMA/DBM/USC-Exos scaffolds

**Figure 3:**
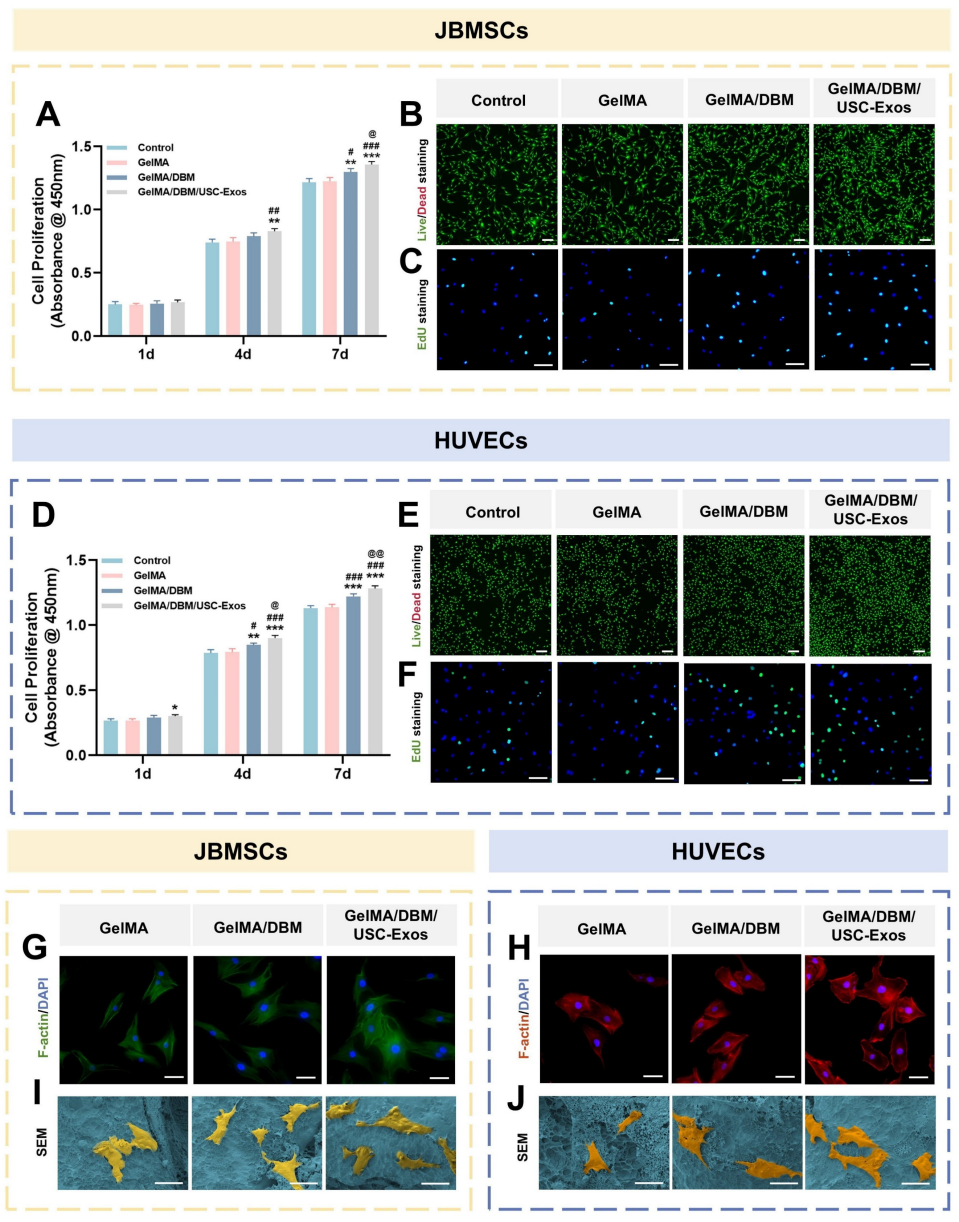
** Cytocompatibility and cell adhesion of GelMA/DBM/USC-Exos scaffolds were evaluated using JBMSCs and HUVECs.** (A, D) CCK-8 assay showing the proliferation of JBMSCs (A) and HUVECs (D) co-cultured with different scaffolds. (B, E) Live/dead staining of JBMSCs (B) and HUVECs (E). Scale bar = 200 μm. (C, F) EdU incorporation assays for JBMSCs (C) and HUVECs (F). Scale bar = 100 μm. (G-J) Immunofluorescence staining of F-actin and SEM images illustrating the cell morphology and adhesion on scaffolds. Scale bar = 50 μm. Data are presented as mean ± SD (n ≥ 3). ^*^ P < 0.05, ^**^ P < 0.01, and ^***^ P < 0.001 versus the control group; ^#^ P < 0.05, ^##^ P < 0.01, and ^###^ P < 0.001 versus the GelMA group; and ^@^ P < 0.05, ^@@^ P < 0.01, and ^@@@^ P < 0.001 versus the GelMA/DBM group.

To evaluate the translational potential of GelMA/DBM/USC-Exos scaffolds in bone tissue engineering, we assessed biocompatibility to support cell adhesion and proliferation using JBMSCs (osteogenesis-related) and HUVECs (angiogenesis-related) 31. CCK-8 assays showed significantly enhanced proliferation of both cell types on GelMA/DBM/USC-Exos scaffolds compared to that on GelMA and GelMA/DBM controls over 7 days, with the most pronounced effects observed after day 4 (Figure 3A and D), indicating that the embedded exosomes retained within the hydrogel matrix exhibited sustained bioactivity capable of promoting continued cell growth. Live/dead staining confirmed high cell viability across all groups, with the GelMA/DBM/USC-Exos scaffold exhibiting the highest density of viable (calcein-positive) cells and an overall viability exceeding 95% (Figure 3B and E). This result supports the conclusion that incorporating DBM and exosomes does not impair cell viability; rather, it synergistically enhances cellular colonization and retention. EdU assays further demonstrated increased DNA synthesis in both JBMSCs and HUVECs cultured on the GelMA/DBM/USC-Exos scaffold (Figure 3C and F), corroborating the CCK-8 results. These findings indicate that an exosome-enriched microenvironment promotes cell proliferation through active mitogenic signaling, supporting its potential to drive *in vivo* tissue regeneration 19.

F-actin immunofluorescence showed that both JBMSCs and HUVECs cultured on GelMA/DBM/USC-Exos scaffolds exhibited well-spread morphologies with distinct stress fibers, indicating robust cytoskeletal organization and strong adhesion (Figure 3G-H). In contrast, the cells grown on pure GelMA appeared rounded with limited spreading. Semi-quantitative analysis confirmed that the spreading area of cells on GelMA/DBM/USC-Exos scaffolds was approximately 2-fold larger than that on GelMA controls (Figure S9). SEM further supported these findings as both cell types displayed extended filopodia and flattened morphologies on the GelMA/DBM/USC-Exos surfaces, indicating firm anchorage and enhanced integrin-mediated adhesion (Figure 3I-J). This improved cell spreading can be attributed to the synergistic effects of DBM-derived biochemical cues and USC-Exos-mediated bioactivity, which collectively enrich the local microenvironment with adhesion molecules, growth factors, and metabolic signals that activate cytoskeletal remodeling. Moreover, the stratified and spatially controlled architecture generated by 3D printing mimics native tissue topography and more effectively facilitates cell adhesion, orientation, and spreading than non-layered or randomly structured scaffolds 8. Because cell morphology is closely correlated with functional outcomes, the observed elongated, spindle-like shapes are associated with improved adhesion, proliferation, and lineage-specific differentiation of osteogenic and endothelial cells 32. These results demonstrate that GelMA/DBM/USC-Exos scaffolds provide a cytocompatible microenvironment that promotes cytoskeletal organization, cell spreading, and dual-lineage adhesion, which are critical features of early-stage osteogenesis and angiogenesis during vascularized bone regeneration.

### *In vitro* immunomodulatory effects of GelMA/DBM/USC-Exos scaffolds

**Figure 4:**
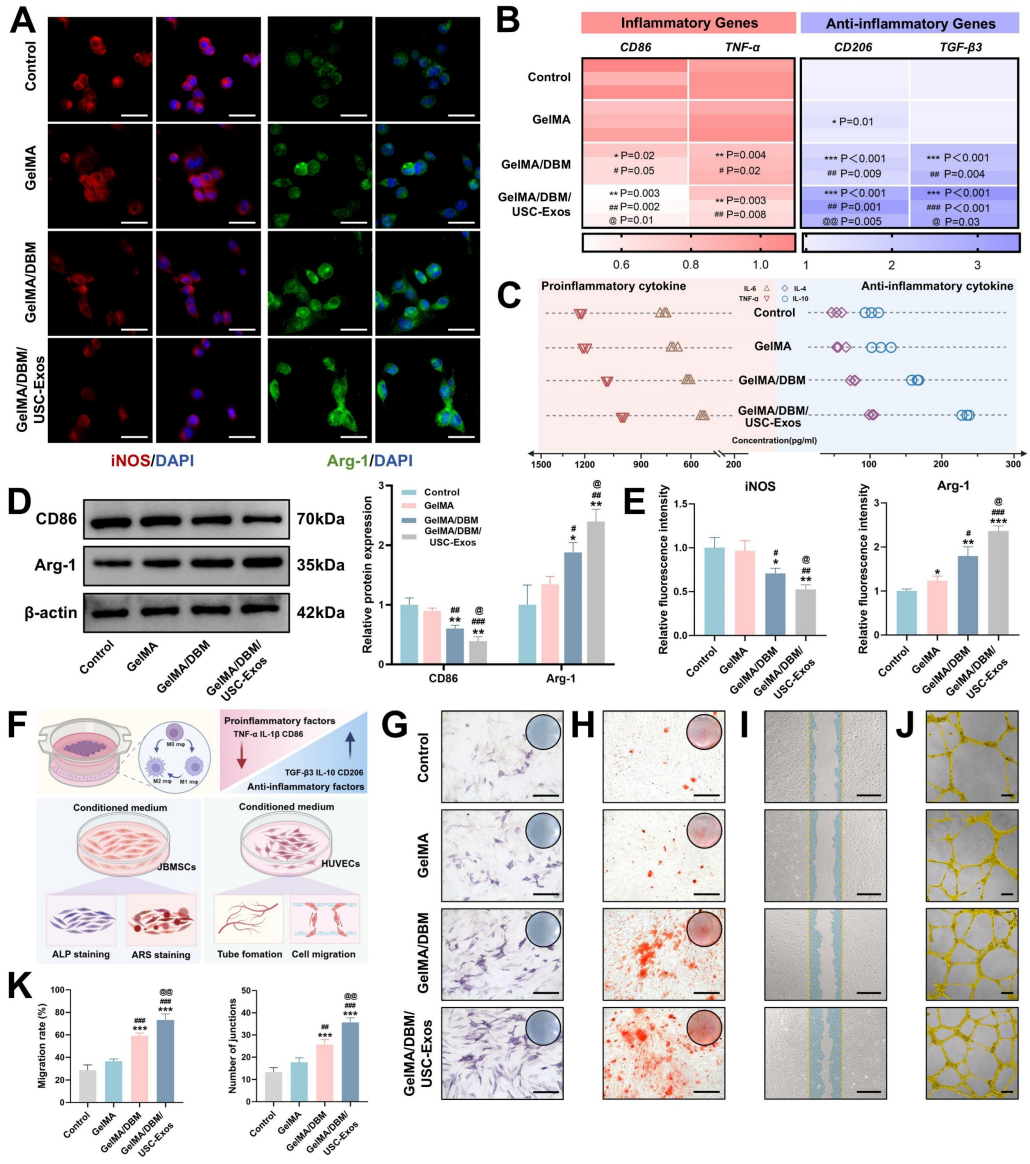
***In vitro* immunomodulatory properties of GelMA/DBM/USC-Exos scaffolds.** (A) Immunofluorescence staining of iNOS and Arg-1 in THP-1 macrophages with quantitative analysis (E). Scale bar = 40 μm. (B) qPCR analysis of pro- and anti-inflammatory gene expression. (C) Cytokine profiles in macrophage-conditioned medium. (D) Western blot analysis of M1/M2 polarization markers. (F) Schematic overview of the experimental setup used to evaluate the effects of macrophage-conditioned medium on angiogenesis and osteogenesis. (G-J) Osteogenic and angiogenic responses of JBMSCs and HUVECs to conditioned medium. Scale bars: 400 μm (G-I) and 100 μm (J). (K) Quantification of tube formation and cell migration. Data are presented as mean ± SD (n ≥ 3). ^*^ P < 0.05, ^**^ P < 0.01, and ^***^ P < 0.001 versus the control group; ^#^ P < 0.05, ^##^ P < 0.01, and ^###^ P < 0.001 versus the GelMA group; and ^@^ P < 0.05, ^@@^ P < 0.01, and ^@@@^ P < 0.001 versus the GelMA/DBM group.

To investigate the immunomodulatory potential of different hydrogel scaffolds, THP-1-derived macrophages were co-cultured with Control, GelMA, GelMA/DBM, or GelMA/DBM/USC-Exos scaffolds in the presence of LPS (1 μg/mL) to mimic an inflammatory environment. Immunostaining showed strong iNOS expression in the Control and GelMA groups, while arginase-1 (Arg-1) was markedly upregulated in the GelMA/DBM/USC-Exos group (Figures 4A and 4E), indicating a shift toward M2 polarization. Consistently, RT-qPCR analysis revealed downregulation of M1 markers (*CD86*, *tumor necrosis factor alpha [TNF-α]*) and upregulation of M2 markers (*CD206*, *transforming growth factor-beta3 [TGF-β3]*), most prominently in the GelMA/DBM/USC-Exos group (Figure 4B). GelMA alone had a minimal effect on M1 marker expression, suggesting a limited immunomodulatory capacity. ELISA results further supported the anti-inflammatory profile, showing decreased IL-6 and TNF-α and increased IL-10 and IL-4 secretion (Figure 4C). Western blot analysis corroborated these findings, showing reduced CD86 and enhanced Arg-1 expression in GelMA/DBM/USC-Exos group (Figure 4D). Although various immune cells participate in modulating the osteogenic microenvironment, macrophages are pivotal regulators of tissue regeneration. After a bone injury, macrophages initially polarize into the proinflammatory M1 phenotype. However, prolonged M1 activation can delay the transition to repair. In contrast, M2 macrophages exert anti-inflammatory and pro-regenerative effects, supporting tissue remodeling and homeostasis 33. Therefore, timely M1-to-M2 repolarization plays a crucial role in shaping downstream cellular responses and determining the outcomes of scaffold integration. Notably, the GelMA/DBM scaffold alone elicited partial M2 polarization, which can be attributed to the intrinsic bioactive cues within DBM, such as bone morphogenetic proteins (BMPs) and ECM-derived cytokines 4,7. However, the incorporation of USC-Exos further amplified this immunomodulatory effect, likely through the exosomal cargo that fine-tunes macrophage signaling and promotes an anti-inflammatory phenotype 18. Thus, rather than acting independently, the DBM and USC-Exos components synergistically established a more pronounced immunoregulatory microenvironment, accelerating the transition from inflammation to tissue repair and ultimately facilitating bone regeneration.

To further elucidate the molecular mechanisms governing macrophage polarization induced by the GelMA/DBM/USC-Exos scaffold, we investigated the involvement of key inflammatory signaling pathways associated with M1/M2 fate determination, particularly the SOCS1/STAT3 and NF-κB pathways. These pathways are widely recognized as central regulators of macrophage inflammatory responses, with SOCS1/STAT3 signaling favoring anti-inflammatory and reparative phenotypes, whereas sustained NF-κB activation drives pro-inflammatory M1 polarization 34,35. Western blot analysis demonstrated significant upregulation of SOCS1 expression and STAT3 phosphorylation, together with marked suppression of NF-κB p65 phosphorylation, in the GelMA/DBM/USC-Exos group (Figure S10). This signaling pattern is characteristic of an anti-inflammatory, pro-regenerative M2 phenotype. It is consistent with the observed reduction in M1-associated markers and pro-inflammatory cytokines, along with enhanced Arg-1 and anti-inflammatory mediator expression. Importantly, this signal reprogramming provides a mechanistic explanation for how USC-Exos functionalization enhances the scaffold's immunomodulatory capacity beyond that of DBM alone.

At the tissue-regeneration level, activation of the SOCS1/STAT3 axis and suppression of NF-κB signaling are particularly relevant because excessive or prolonged NF-κB-driven inflammation impairs angiogenesis, inhibits osteogenic differentiation, and promotes fibrotic tissue formation 35. Conversely, STAT3-driven M2 macrophages secrete pro-angiogenic and osteo-supportive factors that facilitate endothelial cell recruitment, extracellular matrix remodeling, and osteoprogenitor differentiation 34. Therefore, the SOCS1/STAT3-NF-κB signaling balance established by the GelMA/DBM/USC-Exos scaffold provides a mechanistic basis for orchestrating a regenerative immune microenvironment that supports subsequent angiogenesis and bone formation.

Alternatively activated M2 macrophages play key roles in bone regeneration by mediating debris clearance, suppressing inflammation, and promoting angiogenesis and osteogenesis 36. To explore the influence of the hydrogel scaffolds on macrophage polarization and their downstream effects, CMs were collected, as outlined in Figure 4F. *In vitro* assays were used to determine the osteogenic and angiogenic effects of these CM samples. Regarding osteogenesis, CM from the GelMA/DBM/USC-Exos group significantly enhanced JBMSC differentiation, as evidenced by approximately a 3-fold increase in ALP activity and substantially greater calcium deposition compared with the control groups (p < 0.01; Figures 4G-H and S11). The same CM also significantly promoted HUVEC migration and tubulogenesis, resulting in a wound closure rate of 73.32 ± 5.3% and 35.67 ± 2.08 tubular structures per field, both substantially higher than those of the control groups (p < 0.01; Figure 4I-K). These enhanced effects were likely driven by M2 macrophage-derived cytokines in response to the combined impact of USC-Exos and DBM, which promoted a regenerative paracrine environment. Exosomes may also directly contribute to these responses through sustained delivery of bioactive factors. Collectively, these findings highlight that the GelMA/DBM/USC-Exos scaffolds actively modulate immune responses by inducing M2 polarization, thereby creating a pro-regenerative milieu that supports both osteogenesis and angiogenesis, which represent essential processes during the initial stages of bone healing.

### *In vitro* angiogenic and osteogenic properties of GelMA/DBM/USC-Exos scaffolds

**Figure 5:**
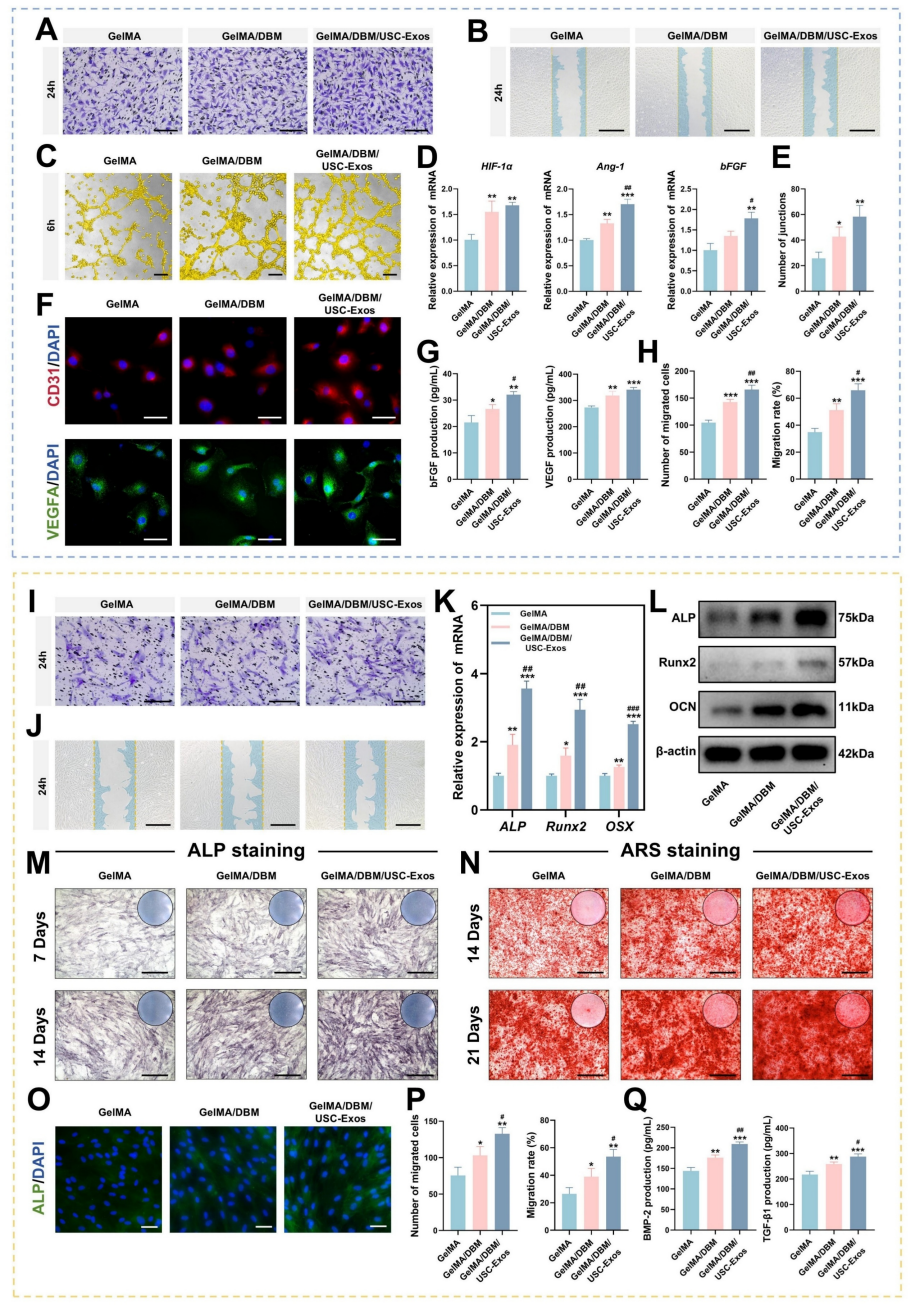
***In vitro* angiogenic and osteogenic effects of GelMA/DBM/USC-Exos hydrogels.** (A-C) Transwell migration, wound healing, and tube formation assays in HUVECs cultured with different scaffolds. Scale bars: 200 μm (A); 400 μm (B); and 100 μm (C). (D) qPCR analysis of angiogenesis-related gene expression. (E) Quantification of tube junctions. (F) Immunofluorescence staining of CD31 and VEGFA. Scale bar = 50 μm. (G) ELISA for VEGF and bFGF secretion. (H) Quantification of migrated cells. (I-J) Transwell migration and wound healing assays with quantitative analysis (P). Scale bars: 200 μm (I) and 400 μm (J). (K-L) qPCR and western blot analysis of osteogenic markers. (M-N) ALP and ARS staining. Scale bar = 400 μm. (O) Immunofluorescence staining for ALP expression. Scale bar = 50 μm. (Q) ELISA of osteogenic cytokines TGF-β1 and BMP-2. Data are presented as mean ± SD (n ≥ 3). ^*^ P < 0.05, ^**^ P < 0.01, and ^***^ P < 0.001 versus the GelMA group; ^#^ P < 0.05, ^##^ P < 0.01, and ^###^ P < 0.001 versus the GelMA/DBM group.

Angiogenesis plays an essential role in bone regeneration by ensuring the continuous supply of oxygen, nutrients, and signaling molecules 37. Therefore, the pro-angiogenic performance of the GelMA/DBM/USC-Exos scaffolds is a critical determinant of their regenerative efficacy. In the Transwell migration assay, the number of migrated HUVECs toward the GelMA/DBM/USC-Exos scaffolds was approximately 1.7-fold higher than that toward GelMA and 1.3-fold higher than that toward GelMA/DBM after 24 h (Figure 5A and H), indicating enhanced chemotactic recruitment mediated by sustained exosome release. Similarly, scratch wound assays revealed the fastest wound closure rate (65.96 ± 4.74%) in the GelMA/DBM/USC-Exos group, compared with 51.22 ± 4.68% for GelMA/DBM and 34.81 ± 2.86% for GelMA alone (p < 0.01; Figure 5B and H). These results suggest that USC-Exos enhanced the pro-angiogenic potential of DBM-containing scaffolds by increasing endothelial cell motility through paracrine signaling, consistent with previous findings in exosome-based hydrogels 17,19.

To further evaluate angiogenic activity, a tube formation assay was performed using Matrigel (Figure 5C). After 6 h, minimal tubular structures were found in the GelMA group, whereas the GelMA/DBM and GelMA/DBM/USC-Exos scaffolds showed extensive network formation (Figure 5E). The GelMA/DBM group exhibited moderate angiogenesis, likely due to its bioactive matrix components. In contrast, the GelMA/DBM/USC-Exos group formed more mature, branched, and interconnected tubules, with a ~2.5-fold increase in junction density compared with the GelMA/DBM scaffold (p < 0.01), indicating that USC-Exos markedly enhanced angiogenic capacity. Immunofluorescence staining revealed elevated CD31 expression after 3 days, with the GelMA/DBM/USC-Exos group exhibiting the highest signal (Figures 5F and S12). These results are consistent with enhanced endothelial activation. Similarly, VEGFA levels were significantly upregulated in the GelMA/DBM/USC-Exos group, followed by those in the GelMA/DBM and GelMA-alone groups (Figures 5F and S12). qPCR further confirmed increased expression of angiogenesis-related genes (Figure 5D), supporting the pro-angiogenic effect of the scaffold. ELISA confirmed elevated secretion of vascular endothelial growth factor (VEGF) and basic fibroblast growth factor (bFGF) in the supernatant of the GelMA/DBM/USC-Exos group (Figure 5G). These findings demonstrate that USC-Exos delivered via a DBM-based matrix retained their angiogenic activity and synergistically enhanced endothelial migration, tube formation, and angiogenic signaling. Consistent with the findings of Fan et al. 19, who incorporated USC-Exos into decellularized porcine small intestinal submucosa hydrogels to promote neovascularization, our results further extend this concept to a bone-specific context. Unlike non-tissue-specific ECM hydrogels derived from soft or visceral tissues, the DBM retains a collagen-rich framework and endogenous matrix-binding domains that can sequester and present proangiogenic cues such as VEGF and bFGF, thereby facilitating endothelial adhesion, migration, and lumen formation.

Moreover, the 3D-printed architecture enables precise spatial control of exosome distribution and release kinetics, ensuring sustained paracrine signaling and continuous stimulation of vascular network formation throughout the construct. These results highlight the therapeutic potential of USC-Exos-functionalized, DBM-integrated 3D-printed scaffolds in promoting vascularization and facilitating tissue regeneration.

As cell recruitment from the surrounding tissue is essential for initiating bone regeneration, Transwell migration and scratch wound healing assays were conducted. The GelMA/DBM/USC-Exos group showed significantly enhanced JBMSC migration compared to that in the GelMA/DBM and GelMA controls (Figure 5I-J and P). After 24 h, this group showed markedly higher cell recruitment and faster wound closure, indicating that exosome incorporation effectively promotes osteoprogenitor cell migration. GelMA/DBM scaffolds outperformed pure GelMA in promoting cell migration, showing approximately 1.3-fold and 1.7-fold increases in the Transwell and scratch assays, respectively (p < 0.01). This is likely due to DBM-derived bioactive matrix cues and chemotactic signals, in line with accumulating evidence that ECM-associated proteins and sGAG are critical for the recruitment of endogenous stem cells 4. Recruiting endogenous stem or progenitor cells is crucial for triggering early-stage tissue repair. These findings suggest that exosome-functionalized, 3D-bioprinted biomimetic hydrogel scaffolds create a pro-regenerative microenvironment conducive to early-stage bone repair.

To assess the osteoinductive capacity of the hydrogels, ALP activity, an early marker of osteogenesis, was evaluated by staining and quantitative analysis. After 7 and 14 days, the GelMA/DBM/USC-Exos group showed the strongest ALP staining (~40% higher positive area), followed by that in the GelMA/DBM and GelMA alone groups (Figures 5M and S13A), indicating enhanced early osteogenic differentiation, likely augmented by the bioactivity of USC-Exos. To assess late-stage differentiation, ARS staining revealed increased calcium deposition in the GelMA/DBM/USC-Exos group on days 14 and 21 (Figure 5N and S13A). Moreover, inclusion of a BMP-2-induced positive control group (100 ng/mL) demonstrated comparable trends in both ALP and ARS assays, further confirming the strong osteoinductive potency of the GelMA/DBM/USC-Exos scaffold (Figures S13C). RT-qPCR analysis revealed that the mRNA levels of *ALP*, *Runt-related transcription factor 2 (Runx2)*, and *Osterix* were markedly elevated in the GelMA/DBM/USC-Exos group, reaching approximately 3-fold higher than those in the GelMA group (Figure 5K). Western blotting confirmed parallel increases in ALP, Runx2, and OCN protein expression (Figure 5L). Immunofluorescence quantification revealed that the ALP fluorescence intensity in the GelMA/DBM/USC-Exos group exhibited a 2.3-fold increase relative to the GelMA group and a 1.2-fold increase compared with GelMA/DBM (Figures 5O and S13B). ELISA results indicated higher secretion of BMP-2 and transforming growth factor beta 1 (TGF-β1), further supporting enhanced osteogenic signaling (Figure 5Q). These results suggest that both biochemical and biophysical cues contributed to the observed enhancement of osteogenic differentiation. Consistent with previous findings using a USC-Exos/GelMA-HAMA/nHAP composite hydrogel 17, USC-Exos similarly promoted osteogenic differentiation by delivering bioactive exosomal cargo that activates osteogenic signaling pathways. However, unlike HAMA/nHAP-based matrices, DBM provided a collagen-rich framework containing endogenous bone morphogenetic proteins, sGAG, and non-collagenous proteins, which can further stimulate osteoprogenitor recruitment and differentiation 4,6. Meanwhile, DBM incorporation increased scaffold stiffness, while the 3D-printed architecture provided precise structural organization and topographical guidance, jointly offering favorable mechanotransductive cues that synergized with USC-Exos-mediated biochemical signaling to potentiate osteogenesis 5. Collectively, these findings indicate that the dual integration of DBM and USC-Exos generates a biomimetic and bioactive scaffold, where structural biomimicry and exosome-driven signaling act in concert to enhance the differentiation of JBMSCs and promote bone tissue regeneration.

Human periodontal ligament stem cells (PDLSCs), a key cell source for alveolar bone regeneration, were utilized to investigate the osteogenic and recruitment capacities of the multifunctional hydrogel due to their multipotency and anatomical proximity to alveolar bone defects 38. The GelMA/DBM/USC-Exos group demonstrated the highest ALP activity on day 7 (44.68 ± 1.51%) and the greatest mineralized nodule formation on day 14 (58.26 ± 2.33%), both significantly higher than those of the other groups (Figure S14A-B and E-F), indicating enhanced osteogenic differentiation. Transwell and scratch assays revealed significantly increased PDLSC migration and wound closure in the exosome-enriched group (Figures S14C-D and G-H), suggesting a strong chemotactic effect. These findings support the hypothesis that the GelMA/DBM/USC-Exos scaffold promotes osteogenesis and cell recruitment, both of which play crucial roles in the progression of bone healing.

### Mechanistic investigation of the osteogenic function of GelMA/DBM/USC-Exos scaffolds

**Figure 6:**
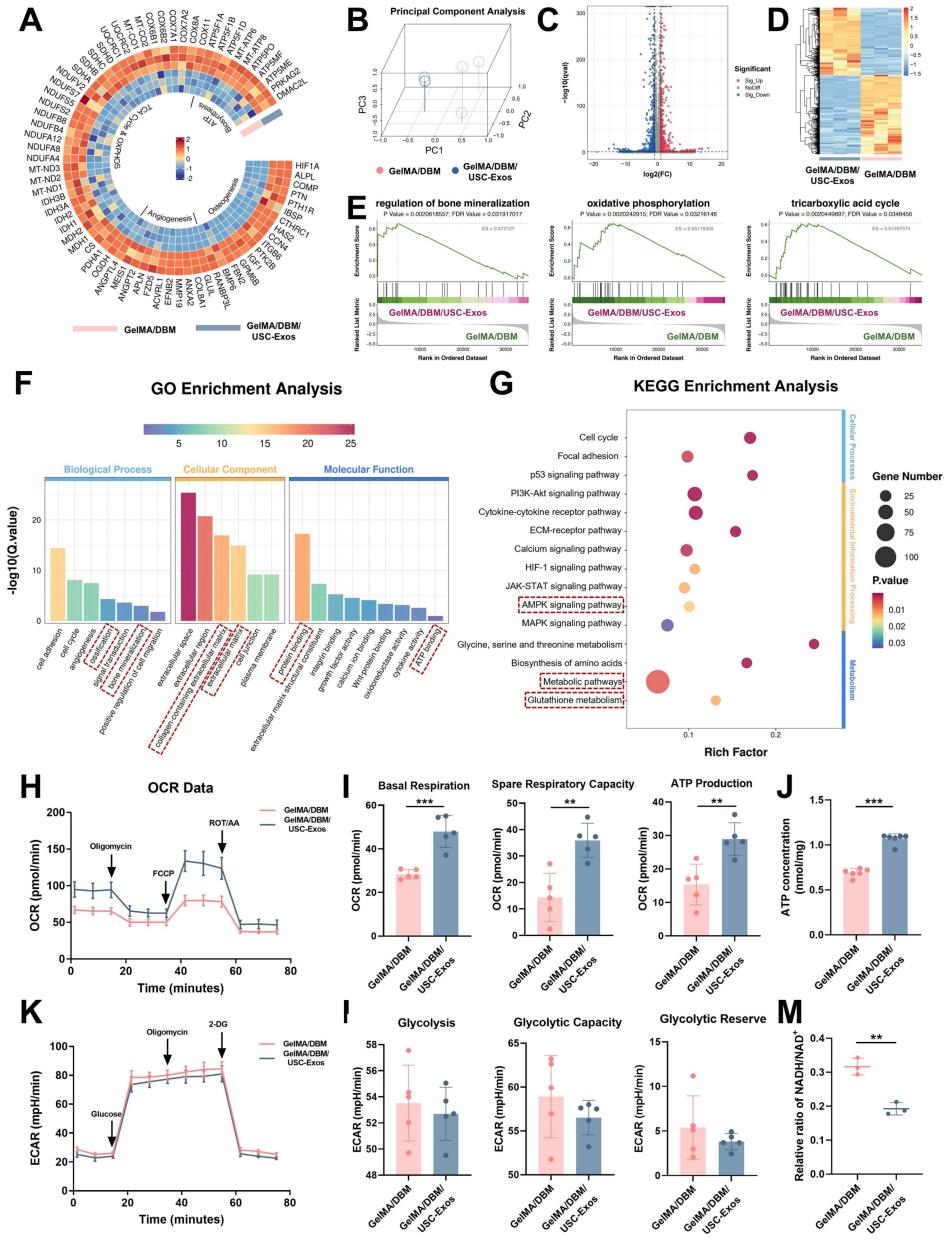
** Transcriptomic analysis reveals that GelMA/DBM/USC-Exos scaffolds regulate osteogenesis through mitochondrial oxidative phosphorylation.** (A) Circular heatmap of DEGs related to osteogenesis, angiogenesis, TCA cycle, OXPHOS, and ATP biosynthesis. (B) Principal component analysis of gene expression profiles comparing GelMA/DBM and GelMA/DBM/USC-Exos groups. (C) Volcano plot of the DEGs. (D) Hierarchical clustering heatmap of gene expression patterns. (E-G) GSEA, Gene Ontology, and KEGG pathway analyses of enriched osteogenic and metabolic pathways. (H-I) Time-resolved OCR curves and quantitative analysis of JBMSCs cultured with GelMA/DBM and GelMA/DBM/USC-Exos scaffolds (n = 5). (J) Intracellular ATP concentration assay between the two groups. (K) ECAR time-course curves of JBMSCs in both groups (n = 5). (L) Quantitative analysis of ECAR-derived parameters, including glycolysis, glycolytic capacity, and glycolytic reserve. (M) Intracellular NADH/NAD⁺ ratio comparison. Data are presented as mean ± SD (n ≥ 3). ^*^ P < 0.05, ^**^ P < 0.01, and ^***^ P < 0.001 versus the GelMA/DBM group.

To elucidate the molecular mechanisms underlying the osteoinductive effects of GelMA/DBM/USC-Exos scaffolds, we performed RNA sequencing on JBMSCs co-cultured with either GelMA/DBM or GelMA/DBM/USC-Exos scaffolds. Principal component analysis revealed a clear separation between groups, indicating significant differences in their transcriptomic profiles (Figure 6B). This was further substantiated by the volcano plot and hierarchical clustering heatmap, where 674 genes were significantly upregulated and 797 genes were downregulated in the GelMA/DBM/USC-Exos group compared with those in the GelMA/DBM group (Figure 6C-D). Gene Ontology enrichment analysis indicated that DEGs were significantly enriched in biological processes closely related to osteogenesis, including cell adhesion, angiogenesis, ossification, and bone mineralization (Figure 6F). In parallel, Kyoto Encyclopedia of Genes and Genomes (KEGG) pathway analysis and Gene Set Enrichment Analysis (GSEA) further highlighted the enrichment in AMPK signaling, glutathione metabolism, and pathways related to the TCA cycle and OXPHOS (Figures 6E and G, and S15). Notably, genes regulating mitochondrial bioenergetics and osteogenic/angiogenic processes were markedly upregulated in the exosome-treated group (Figure 6A), which was further confirmed using qPCR (Figure S16A-B). Collectively, these transcriptomic insights suggested that the enhanced osteoinductive effect of the GelMA/DBM/USC-Exos scaffolds was tightly linked to the activation of mitochondrial OXPHOS, modulation of glutathione metabolism, and upregulation of AMPK signaling. These pathways likely converge to optimize the bioenergetic and redox states of JBMSCs, thereby facilitating their osteogenic differentiation.

Glycolysis and OXPHOS are the two main metabolic pathways supporting ATP production during BMSC differentiation 39,40. Although BMSCs primarily rely on glycolysis under basal conditions, osteoinductive cues trigger a metabolic shift toward mitochondrial OXPHOS to meet the sustained energy demands of osteogenesis 40. The OCR was assessed as a direct indicator of mitochondrial respiratory capacity. The GelMA/DBM/USC-Exos group showed significantly elevated mitochondrial respiration (Figure 6H-I), indicating enhanced OXPHOS. In contrast, the ECAR, an index of glycolytic flux, remained unchanged across groups (Figures 6K-L), suggesting minimal glycolytic involvement. Luminescence-based ATP assays confirmed the significantly higher ATP levels in the exosome-treated group (Figure 6J), which aligned with the transcriptomic data. A reduced intracellular NADH/NAD⁺ ratio (Figure 6M) further supported the enhanced electron transport chain activity. These results demonstrated that the GelMA/DBM/USC-Exos scaffolds significantly boosted mitochondrial functionality and oxidative metabolism in JBMSCs. By increasing the generation of OCR and ATP, this metabolic reprogramming ensures sufficient bioenergetic support for osteogenic differentiation, thereby underpinning the efficacy of the scaffold in bone regeneration.

**Figure 7:**
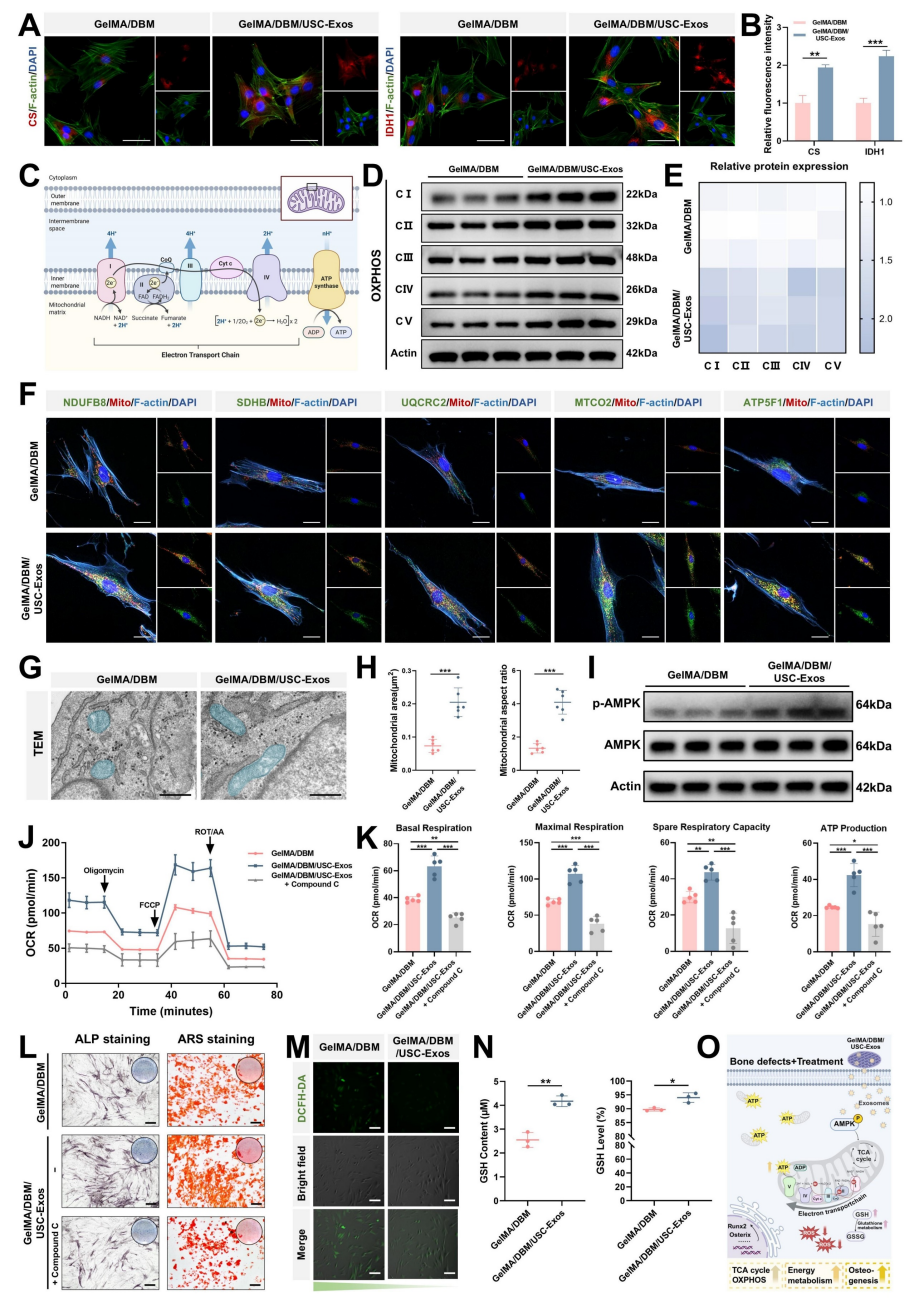
** Osteogenic mechanism in GelMA/DBM/USC-Exos scaffolds.** (A-B) Immunofluorescence staining and fluorescence quantification of CS and IDH1. Scale bar = 50 μm. (C) Schematic of mitochondrial oxidative phosphorylation via the electron transport chain. (D-E) Western blot analysis of electron transport chain complex subunits. (F) Immunofluorescence staining of electron transport chain complex subunits. Scale bar = 20 μm. (G-H) TEM images and morphological quantification of mitochondria. Scale bar = 500 nm. (I) Western blot analysis of AMPK signaling pathway proteins. (J-K) OCR time-course and quantitative analysis (n = 5). (L) ALP and ARS staining after AMPK inhibition. Scale bar = 200 μm. (M-N) DCFH-DA staining of intracellular ROS and quantification of GSH levels. Scale bar = 100 μm. (O) Proposed mechanism of GelMA/DBM/USC-Exos-induced osteogenic differentiation in JBMSCs. Data are presented as mean ± SD (n ≥ 3). ^*^ P < 0.05, ^**^ P < 0.01, and ^***^ P < 0.001 versus the GelMA/DBM group.

To further explore the mechanisms underlying the TCA cycle and OXPHOS upregulation, we analyzed the expression of essential metabolic enzymes and mitochondrial components. CS and IDH1, two rate-limiting enzymes in the TCA cycle, showed markedly increased protein expression in the GelMA/DBM/USC-Exos group, as confirmed by immunofluorescence staining (Figure 7A-B), indicating enhanced mitochondrial metabolic activity. Given that electron transport chain complexes are embedded within the inner mitochondrial membrane and are critical for driving OXPHOS (Figure 7C), western blot and immunofluorescence analyses revealed elevated expression of complexes I-V in the exosome-treated group compared with that in the GelMA/DBM controls (Figure 7D-F). To further validate these observations, we examined the mitochondrial ultrastructures using TEM (Figure 7G). JBMSCs treated with GelMA/DBM/USC-Exos scaffolds exhibited a significantly greater mitochondrial area and aspect ratio than those in the control group (Figure 7H), indicating increased mitochondrial elongation and fusion. These morphological changes reflect improved mitochondrial dynamics and an expanded inner membrane surface area, supporting elevated OXPHOS activity 41. Consistently, treatment with mitochondrial inhibitors, oligomycin or rotenone, significantly reduced ALP activity in scaffold-treated JBMSCs, indicating that mitochondrial respiration is required for scaffold-induced osteogenic differentiation (Figure S17A-B). Collectively, these findings strongly support the conclusion that GelMA/DBM/USC-Exos scaffolds enhance mitochondrial metabolic capacity, driving increased oxidative metabolism that supports osteogenic differentiation.

AMPK signaling is crucial in transcriptional regulation, cell proliferation, osteogenesis, angiogenesis, and metabolic homeostasis, particularly in OXPHOS modulation 42. KEGG analysis revealed significant enrichment of the AMPK pathway in the GelMA/DBM/USC-Exos group, suggesting its potential involvement in observed metabolic reprogramming. To verify AMPK activation, western blot analysis was performed, which demonstrated increased AMPK phosphorylation upon scaffold stimulation (Figure 7I), indicating pathway activation and its potential role in enhancing TCA cycle activity and mitochondrial respiration. Based on these observations, we hypothesized that GelMA/DBM/USC-Exos scaffolds promote energy metabolism in JBMSCs by activating AMPK, thereby enhancing mitochondrial respiration and ATP production to support osteogenesis. To further validate this hypothesis, we used Compound C, a selective AMPK inhibitor (Figure S18A) 43. Seahorse analysis showed that AMPK inhibition significantly reduced the OCR in JBMSCs co-cultured with the GelMA/DBM/USC-Exos scaffolds (Figure 7J-K). NAD⁺/NADH ratios also decreased (Figure S18B), confirming suppressed mitochondrial function. These results suggested that AMPK inhibition attenuated the scaffold-induced enhancement of mitochondrial OXPHOS and overall respiratory activity. Furthermore, inhibition of AMPK markedly reduced ALP activity and mineralized nodule formation (Figures 7L and S18C), indicating suppression of osteogenic differentiation. These results demonstrate that AMPK activation is not merely associated with, but is causally required for, the scaffold-induced enhancement of mitochondrial OXPHOS and osteogenic activity. Previous studies have shown that the AMPK-OXPHOS axis acts as a metabolic driver of osteogenesis by coupling mitochondrial oxidative activity to the bioenergetic demands of differentiating osteoblasts 44-46. However, most of these findings were correlative, and direct mechanistic validation within exosome-functionalized biomaterials has not been reported. Building on these findings, our study provides explicit causal evidence that USC-Exos incorporated into GelMA/DBM scaffolds activate AMPK signaling, which in turn elevates TCA cycle flux and OXPHOS efficiency, thereby creating an energy-enriched microenvironment that promotes osteogenic differentiation.

ATP, the main cellular “bioenergy currency” in mammals, is essential for regulating stem cell proliferation and differentiation. Recent investigations have emphasized the importance of metabolic reprogramming as a pivotal mechanism in tissue regeneration, with exosomes emerging as critical modulators of cellular bioenergetics 12-14,40. Mechanistically, exosomes carry mitochondrial components, such as ATP synthase, mitochondrial transcription factor A, and other mitochondria-derived proteins or nucleic acids, thereby enhancing mitochondrial function and improving the efficiency of cellular energy production and utilization 13. Moreover, exosomes have been shown to interact with and regulate several key signaling pathways and biological processes involved in cellular energy metabolism in bone cells, including AMPK, autophagy, and ROS pathways 14,47. Thus, interest in exosomes as a novel class of bioenergetically active factors has notably increased. Building on this concept, our research demonstrates that USC-Exos, when incorporated into GelMA/DBM scaffolds, promote the osteogenic differentiation of JBMSCs by enhancing the TCA cycle and OXPHOS through AMPK pathway activation. Notably, USC-Exos promoted osteogenesis by enhancing mitochondrial OXPHOS, a mechanism not previously demonstrated in bone regeneration. This metabolic upregulation facilitates the creation of an energy-rich microenvironment that supports bone regeneration. Collectively, these findings revealed that USC-Exos function not only as bioactive cues but also as metabolic modulators, driving AMPK-dependent mitochondrial activation and reprogramming cellular energy metabolism.

Intracellular ROS play a dual role in regulating MSC fate, with moderate levels promoting osteogenic differentiation of MSCs, while excessive accumulation impairs osteogenesis and promotes adipogenesis 48. Therefore, maintaining redox balance is essential for promoting effective bone regeneration. In this study, the bioenergetic enhancement induced by the GelMA/DBM/USC-Exos scaffolds was associated with an upregulation of mitochondrial respiration and electron transport activity. Under physiological conditions, this is typically associated with increased ROS production, which is generally detrimental to osteogenesis. However, quantitative fluorescence analysis indicated a significant decrease in intracellular ROS accumulation in the GelMA/DBM/USC-Exos group compared with the GelMA/DBM group (Figure 7M). This suggests that, despite elevated OXPHOS, oxidative stress was effectively mitigated, thereby preserving a redox microenvironment favorable for osteogenic differentiation. KEGG and GSEA revealed significant enrichment of the glutathione metabolism pathway in USC-Exo-treated cells, highlighting the key role of this antioxidant system in maintaining redox balance 49. Based on these findings, we propose that the scaffold enhances glutathione-dependent ROS detoxification and maintains optimal ROS levels, thereby supporting osteogenic signaling. Supporting this hypothesis, the intracellular levels of GSH, a key antioxidant derived from glutamine metabolism 50, were markedly increased in the USC-Exos group (Figure 7N). Collectively, these results suggest that USC-Exos-loaded scaffold facilitates glutathione-mediated ROS clearance, thereby sustaining a redox environment conducive to osteogenic differentiation and validating the role of enhanced glutathione metabolism in ROS regulation under osteogenic conditions.

Collectively, the GelMA/DBM/USC-Exos scaffold promotes bone regeneration through a synergistic dual mechanism. It activates AMPK signaling to enhance mitochondrial ATP production and oxidative metabolism while simultaneously upregulating glutathione-mediated ROS clearance to maintain redox homeostasis. This coordinated interplay establishes a metabolically supportive and oxidative stress-regulated microenvironment, thereby promoting osteogenic gene expression and JBMSC differentiation (Figure 7O), ultimately contributing to effective bone regeneration.

### *In vivo* evaluation of immunomodulation, angiogenesis, and bone regeneration in a rat alveolar bone defect model

**Figure 8:**
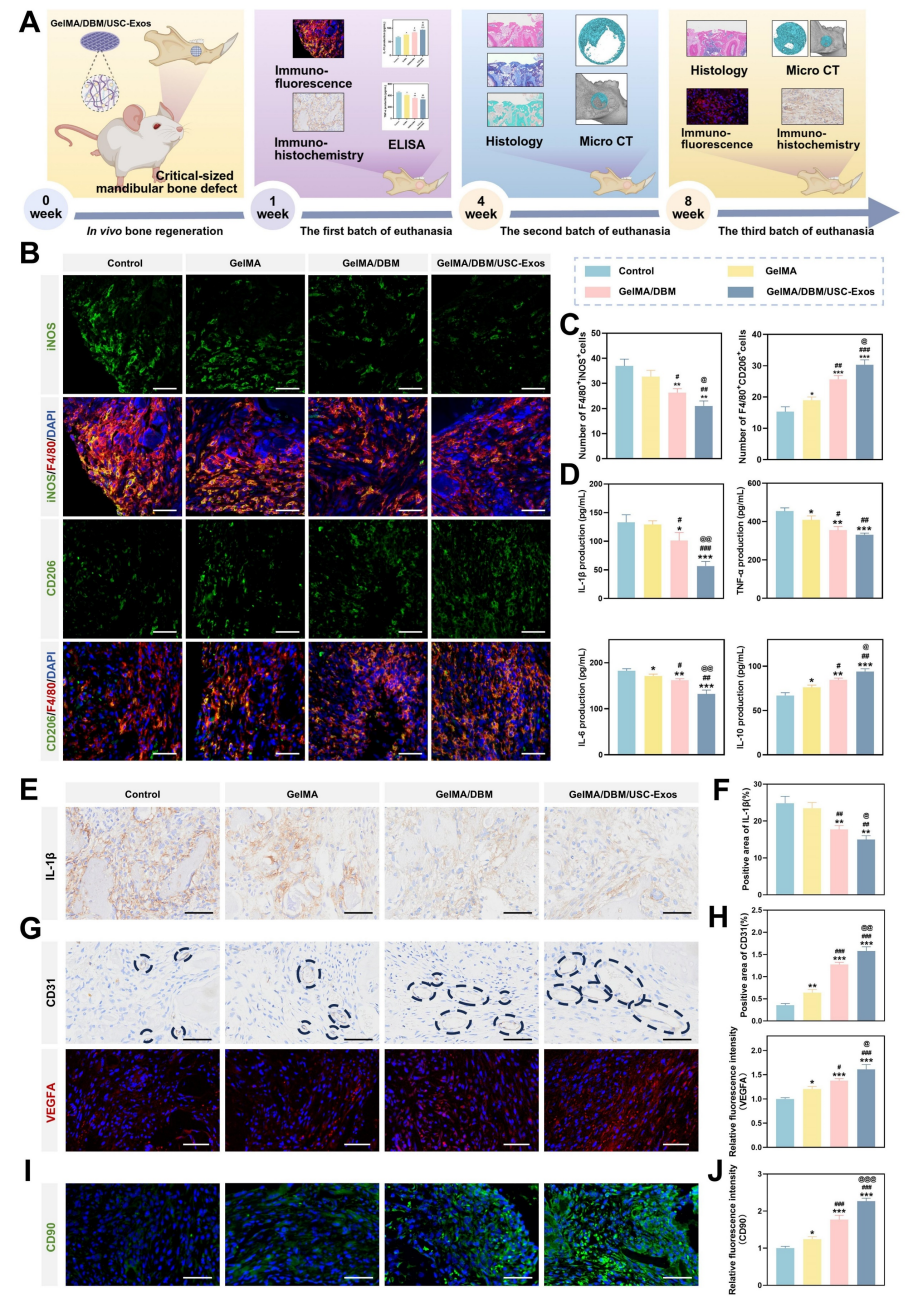
** GelMA/DBM/USC-Exos scaffolds induce *in vivo* immunomodulatory effects, neovascularization, and stem cell recruitment in a mandibular defect model.** (A) Schematic timeline of scaffold implantation, animal euthanasia, and analysis time points. (B) Immunofluorescence staining of iNOS/F4/80 and CD206/F4/80 to identify M1 and M2 macrophages. Scale bar = 40 μm. (C) Quantification of M1/M2 macrophage infiltration. (D) ELISA results for cytokines at the defect sites. (E-F) IL-1β immunohistochemistry and the corresponding quantification. Scale bar = 50 μm. (G-H) CD31 and VEGFA immunostaining and quantification for neovascularization assessment. Scale bar = 50 μm. (I-J) CD90 immunofluorescence and quantification to evaluate MSC recruitment. Scale bar = 50 μm. Data are presented as mean ± SD (n ≥ 3). ^*^ P < 0.05, ^**^ P < 0.01, and ^***^ P < 0.001 versus the control group; ^#^ P < 0.05, ^##^ P < 0.01, and ^###^ P < 0.001 versus the GelMA group; and ^@^ P < 0.05, ^@@^ P < 0.01, and ^@@@^ P < 0.001 versus the GelMA/DBM group.

*In vitro* studies showed that the GelMA/DBM/USC-Exos hydrogel exhibited strong osteogenic, angiogenic, and immunomodulatory properties, indicating its high potential for bone regeneration. To validate these effects *in vivo*, the ability of the scaffolds to modulate the regenerative microenvironment and promote bone repair was assessed, with untreated defects serving as controls (Figures 8A and S19). H&E staining of the major organs showed no histopathological abnormalities, and hemolysis assays revealed negligible hemolytic activity (Figures S20A-B and 21A-B), collectively confirming the systemic biocompatibility and safety of the scaffold.

Post-injury, early-stage inflammation has a critical influence on bone healing by directing the recruitment of immune cells and local immune responses 33,36. To evaluate *in vivo* immunomodulation, macrophage polarization was examined one-week post-implantation using immunofluorescence. Macrophages expressing iNOS and CD206 are marked as M1 (pro-inflammatory) and M2 (anti-inflammatory) macrophages, respectively. The control group exhibited high F4/80⁺iNOS⁺ macrophage levels, indicating robust inflammation, whereas all the scaffold-treated groups showed reduced M1 macrophages and increased F4/80⁺CD206⁺ M2 macrophages. Notably, the GelMA/DBM/USC-Exos group showed the most pronounced M2 shift, approximately 2-fold greater than that of the control group (p < 0.01; Figure 8B-C). To further assess inflammation, IL-1β expression was measured on day 7. The control group showed the highest levels (24.83 ± 1.84%), whereas both the GelMA/DBM (17.72 ± 1.03%) and GelMA/DBM/USC-Exos groups (14.97 ± 1.06%) exhibited significant reductions, with the latter showing the greatest level of suppression (Figure 8E-F). Consistently, ELISA results revealed decreased IL-1β, TNF-α, and IL-6, alongside increased IL-10 in the GelMA/DBM/USC-Exos group (Figure 8D). Collectively, these findings confirmed that the GelMA/DBM/USC-Exos scaffold, through the combined effects of DBM and exosomes, attenuated early inflammation by shifting macrophage polarization from the M1 to M2 phenotype, thereby fostering a pro-regenerative immune environment conducive to bone repair.

Angiogenesis is critical for bone regeneration because neovascularization enhances cellular activity, recruits MSCs to the defect sites, and supports ECM synthesis during healing 37. *In vivo* evaluation revealed that defects treated with the GelMA/DBM/USC-Exos scaffold exhibited approximately 2-3-fold larger CD31⁺ vascularized areas than those in defects treated with other hydrogel formulations, indicating abundant blood vessel infiltration (Figure 8G-H). This was further supported by the markedly elevated VEGFA expression in the GelMA/DBM/USC-Exos group, confirming its strong pro-angiogenic potential. This angiogenic boost was attributed to the synergistic action of USC-derived exosomes and DBM within the GelMA/DBM/USC-Exos scaffold. Specifically, improved vascularization in the USC-Exos-loaded scaffold group was associated with significantly enhanced bone formation compared to that in the GelMA/DBM and GelMA-alone groups. This can be explained by the functionalization imparted by USC-Exos, which effectively promotes endothelial cell migration, proliferation, and tube formation when incorporated into various biomaterials 17,19. These results emphasize the therapeutic promise of the GelMA/DBM/USC-Exos hydrogel as a bioactive, controlled-release platform that effectively promotes vascularization in alveolar bone defects.

Effective bone regeneration depends on the recruitment of endogenous stem cells and subsequent differentiation into osteoblasts 51. Immunofluorescence staining for CD90, an MSC marker, was performed to assess stem cell homing (Figure 8I-J). Minimal CD90⁺ signals were detected in the control group, indicating minimal poor stem cell accumulation. In contrast, both GelMA/DBM and GelMA/DBM/USC-Exos groups exhibited markedly higher CD90⁺ signal intensity, with the latter showing the most robust expression. This enhanced recruitment was attributed to the favorable microstructure enabled by the precision of 3D printing, which facilitated the synergistic effects between the osteoconductive properties of DBM and the bioactivity of USC-derived exosomes, in accordance with previous findings 5,17. These results underscore the critical role of the GelMA/DBM/USC-Exos scaffold in promoting stem cell homing and early cellular colonization at the defect site, which are the key steps for successful osseointegration and bone regeneration.

**Figure 9:**
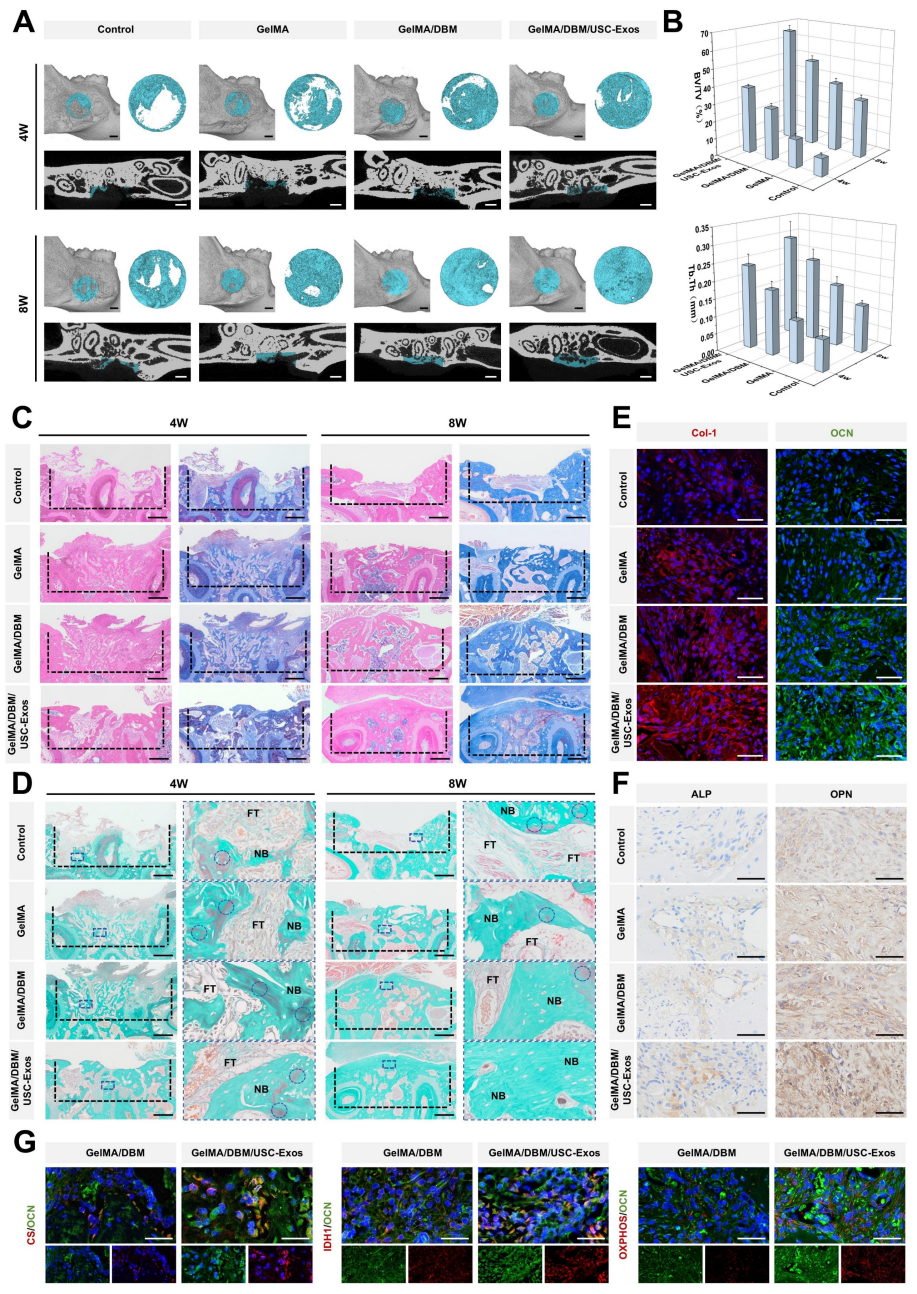
** Comprehensive evaluation of bone regeneration using micro-CT imaging and histological analysis.** (A) 3D reconstructions and sagittal/axial micro-CT images. Scale bar = 1 mm. (B) Quantitative analysis of (BV/TV) and (Tb.Th). (C, D) Histological staining of the regenerated bone. FT, fibrous tissue; NB, newly formed bone; and blue circle, osteoid. Scale bar = 500 μm. (E) COL-1 and OCN immunofluorescence staining of defect regions. Scale bar = 50 μm. (F) ALP and OPN immunohistochemical staining for osteogenic activity assessment. Scale bar = 50 μm. (G) Immunofluorescence staining analysis of OCN with key metabolic enzymes. Scale bar = 40 μm. Data are presented as mean ± SD (n ≥ 3).

Micro-CT analysis revealed minimal peripheral bone formation in the control defects at both time points. Conversely, the GelMA/DBM and GelMA/DBM/USC-Exos groups showed substantial bone growth after 4 weeks, evolving into mature, contiguous structures by week 8 (Figure 9A). The moderate improvement in the GelMA group compared with the control group likely stemmed from the inherent bioactivity and ECM mimicry of GelMA, consistent with previous findings 52. Quantitative micro-CT analysis confirmed these observations, highlighting the superior bone regeneration in the GelMA/DBM/USC-Exos group. At 8 weeks, the bone volume/total volume ratio (BV/TV) reached 65.61 ± 3.02%, significantly exceeding that of the GelMA/DBM (49.97 ± 2.70%), GelMA (39.44 ± 2.59%), and control (32.94 ± 2.43%) groups (Figure 9B). Other key structural parameters, including increased trabecular thickness (0.29 ± 0.05 mm) and decreased trabecular separation (0.132 ± 0.01 mm⁻¹), further supported enhanced bone density and organization (Figures 9B and S22). These results demonstrated the efficacy of the GelMA/DBM/USC-Exos scaffold in promoting rapid biominerallization and robust bone regeneration, making it a promising strategy for treating alveolar defects.

Histological staining was performed to assess the structural and qualitative features of the regenerated bone. At both 4 and 8 weeks, the control group sections showed sparse fibrous tissue at the defect margins, indicating poor regeneration, which is consistent with the micro-CT findings. In contrast, the GelMA/DBM and GelMA/DBM/USC-Exos groups showed robust lamellar bone formation, with the latter exhibiting near-complete defect closure and a mature bone structure after 8 weeks (Figure 9C). Masson's trichrome staining confirmed the presence of abundant organized collagen in the GelMA/DBM/USC-Exos group, whereas the control and GelMA groups showed disorganized deposition. Goldner's trichrome staining revealed significantly more mature lamellar bone and reduced osteoids in the GelMA/DBM/USC-Exos group, suggesting effective mineralization (Figure 9D). Bone formation advanced centripetally, indicating that the scaffold guided osteoinduction and osteoconduction by supporting osteoprogenitor migration and differentiation. Consistently, nanoindentation testing revealed that the regenerated bone in the GelMA/DBM/USC-Exos group exhibited a markedly higher elastic modulus (9.11 ± 1.22 GPa) and hardness (0.28 ± 0.03 GPa) compared with the other groups (Figure S23A-B). These values are comparable to those of native trabecular bone reported in previous studies 53,54, suggesting that the regenerated tissue possesses near-physiological stiffness and strength. Due to the presence of diverse biological and biochemical cues, hydrogels incorporating DBM more effectively mimic the native 3D tissue architecture and demonstrate a favorable degradation profile, thereby enhancing cell infiltration and tissue ingrowth 4,6,55. In addition, functionalization with USC-Exos introduced an additional regenerative capability. The integration of these two components into a 3D-printed hydrogel scaffold results in a bioactive construct that significantly promotes bone tissue regeneration through both early ECM deposition and sustained mineralization over time.

To further investigate the *in vivo* osteogenic and biomineralization capabilities of the multifunctional hydrogel, immunohistochemical and immunofluorescence staining were performed to detect key markers, including COL-1, OCN, ALP, and OPN. Both the GelMA/DBM and GelMA/DBM/USC-Exos groups showed markedly elevated expression compared to that in the controls, with the GelMA/DBM/USC-Exos group exhibiting the most extensive staining, indicating enhanced matrix maturation and mineralization (Figures 9E-F and S24A-B). Moreover, immunostaining confirmed activation of the AMPK pathway *in vivo*, consistent with the *in vitro* results, indicating that the AMPK signaling axis was effectively triggered within the regenerated bone tissue (Figure S25). Additionally, co-localization studies were performed using OCN in conjunction with the key TCA cycle enzymes, CS and IDH1, to explore the metabolic landscape associated with osteogenesis (Figure 9G).

Regions displaying strong OCN positivity also exhibited high levels of CS and IDH1 expression, whereas areas lacking OCN expression showed minimal expression of these metabolic enzymes. The results revealed a substantial spatial overlap in the GelMA/DBM/USC-Exos group, suggesting a link between oxidative metabolism and active bone formation. Further, OXPHOS-related protein staining confirmed the increased mitochondrial activity and a higher proportion of OXPHOS-positive cells in the GelMA/DBM/USC-Exo group. Taken together, these findings provide *in vivo* evidence that the GelMA/DBM/USC-Exos scaffold activates AMPK signaling, enhances mitochondrial oxidative metabolism, and promotes the expression of downstream osteogenic markers, thereby supporting matrix synthesis and mineralization. These results highlight the role of exosomes as potent regulators of cellular bioenergetics during bone tissue regeneration 12-14,47,56.

### Shape-adaptive bone regeneration using customized 3D-printed scaffolds

**Figure 10:**
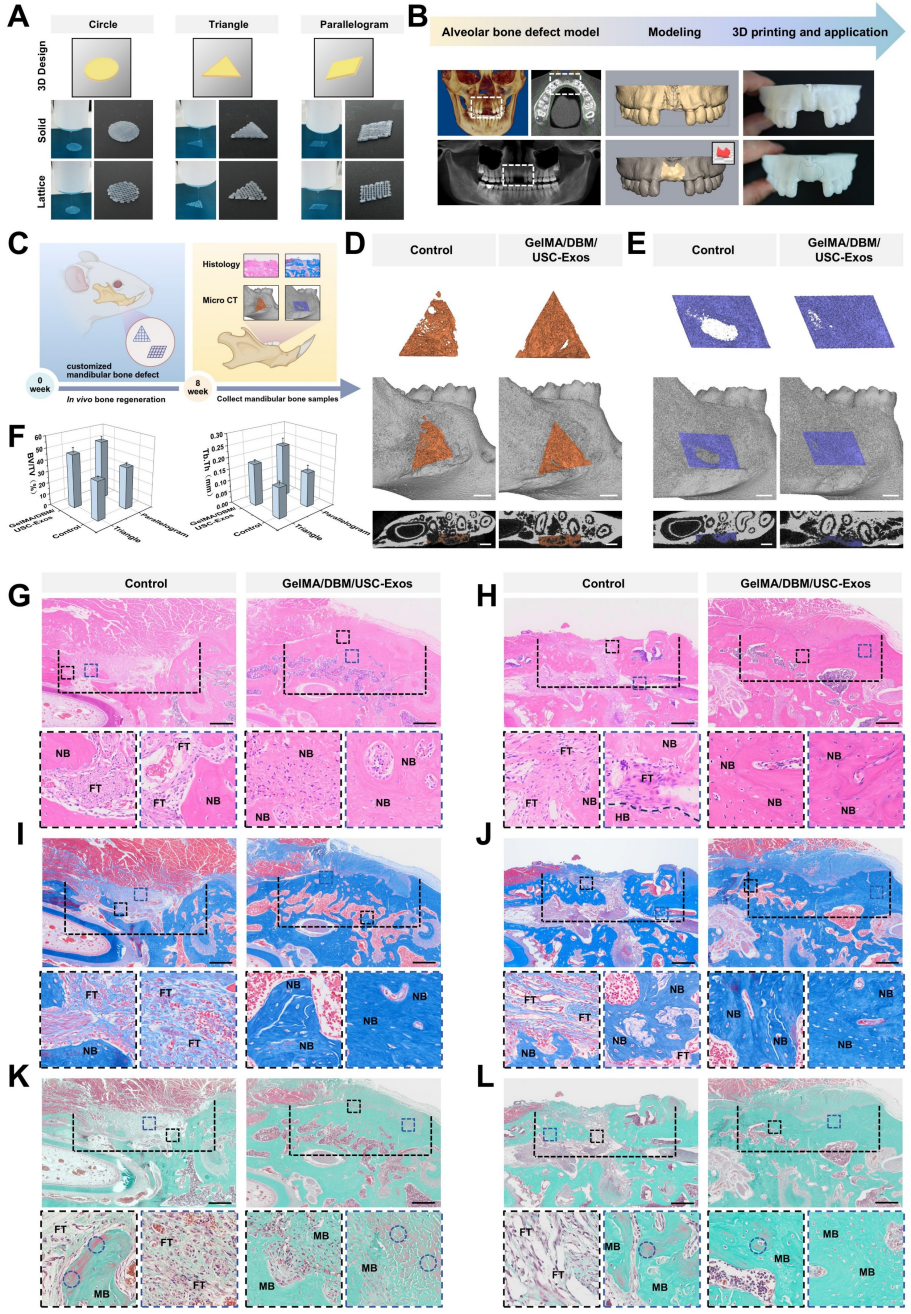
** Geometric adaptability of 3D-printed GelMA/DBM/USC-Exos scaffolds for alveolar bone regeneration.** (A) Representative images of 3D-printed hydrogel scaffolds with varied geometries and internal architectures. (B) Digital modeling of mandibular defects based on clinical imaging for personalized scaffold design. (C) Schematic of the geometry-matching strategy and implantation procedure. (D-E) Micro-CT reconstructions of triangular and parallelogram-shaped defects. Scale bar = 1 mm. (F) Quantitative morphometric analysis of the defect regions. (G-L) Histological evaluation using H&E, Masson's trichrome, and Goldner's trichrome staining. FT, fibrous tissue; NB, newly formed bone; HB, host bone; MB, mineralized bone; and blue circle, osteoid (immature bone). Scale bar = 500 μm. Data are presented as mean ± SD (n ≥ 3).

Building on its efficacy in critically sized circular defects, we further explored the GelMA/DBM/USC-Exos scaffold for shape-specific reconstruction of the alveolar ridge. To assess its clinical adaptability, the printability and structural fidelity of the bioink were evaluated. As shown in Figure 10A, the hydrogel maintained high geometric precision across various complex shapes (circular, triangular, and parallelogram) in both the solid and lattice forms. This confirmed its excellent moldability and suitability for replicating irregular alveolar bone contours.

Customizing scaffolds to match patient-specific defect geometries is a key advancement in regenerative medicine. Using cone beam computed tomography data from a patient with anterior alveolar bone loss, a digital model of the defect was reconstructed, enabling the 3D printing of a personalized GelMA/DBM/USC-Exos scaffold (Figure 10B). This process demonstrates the anatomical adaptability of the scaffold and its potential for clinical translation through the integration of digital planning and biofabrication. This workflow affirms the feasibility of applying patient-specific imaging to guide precise, intraoperative scaffold design for alveolar ridge augmentation.

To evaluate the bone-regenerative capacity of personalized 3D-printed scaffolds under shape-specific conditions, triangular and parallelogram-shaped critical-sized defects were created in rat mandibles (Figures 10C and S19). At eight weeks post-implantation, micro-CT revealed significantly enhanced bone formation in defects treated with GelMA/DBM/USC-Exos scaffolds compared to the controls, as supported by the higher BV/TV and Tb.Th. values (Figure 10D-F). Histological analyses further confirmed robust new bone formation, including mature trabeculae, development of the marrow cavity, and a well-organized collagen architecture in the treated groups (Figure 10G-L), indicating active bone remodeling. These findings demonstrate that the 3D-printed GelMA/DBM/USC-Exos scaffold supports precise, geometry-adaptive bone regeneration while preserving its bioactivity. Overall, this study highlights the often-overlooked impact of scaffold geometry on bone tissue engineering and shows that bioactive 3D-printable hydrogels can effectively integrate mechanical customization with biological performance, thereby advancing their clinical translational potential.

Taken together, this study establishes a multifunctional 3D-printed GelMA/DBM/USC-Exos scaffold that effectively promotes bone regeneration; however, several limitations should be acknowledged. The mechanisms through which the scaffold regulates macrophage polarization and vascularization remain insufficiently understood, and the causal relationship between angiogenesis and osteogenesis requires further investigation. Future studies will focus on identifying key ECM components and signaling pathways involved in macrophage-mediated immunomodulation and vascular remodeling, supported by vascular inhibition and *in vivo* imaging models to elucidate the dynamic interplay between angiogenesis and osteogenesis. Moreover, the present findings were obtained in a small-animal model with a relatively short observation period, which may not fully replicate the biomechanical and immunological complexity of human alveolar bone; therefore, validation in large-animal models with longer follow-up will be necessary to assess long-term regenerative stability and translational feasibility. In addition, the large-scale production, standardized isolation, and stability of USC-Exos remain significant challenges for clinical translation. Future efforts will focus on developing scalable exosome biomanufacturing and advanced biofabrication strategies to enhance the reproducibility, functionality, and translational potential of personalized regenerative therapies.

## Conclusions

Inspired by the structure of a flowerbed, we developed a biomimetic 3D-printed hydrogel scaffold composed of GelMA and DBM, which was functionally enhanced using USC-Exos. A bone microenvironment-matched scaffold was engineered through machine-learning-guided optimization and exhibited favorable printability, mechanical integrity, and sustained exosome release capacity. *In vitro* assessments demonstrated that the scaffold displayed robust immunomodulatory, angiogenic, and osteoinductive properties. *In vivo*, the scaffolds facilitated robust alveolar bone regeneration in rat models of critical-sized mandibular defects with varying shapes and complexities. Transcriptomic profiling revealed that GelMA/DBM/USC-Exos scaffolds markedly reprogrammed the energy metabolism of JBMSCs, driving osteogenic differentiation. This effect was evidenced by the significant upregulation of OXPHOS and TCA cycle activity, which was mediated by the activation of the AMPK signaling pathway. Overall, our findings present a biomimetic scaffold with structure-function compatibility tailored to the osteogenic microenvironment, achieved by integrating tissue-specific DBM-based architecture and USC-Exos-mediated bioactivity within a 3D bioprinting framework. Benefiting from synergistic biophysical and biochemical cues, this multifunctional platform functions as a metabolically activated construct, holding significant promise as a next-generation, cell-free implant for personalized bone regeneration.

## Supplementary Material

Supplementary methods and figures.

## Abbreviations

ALP: alkaline phosphatase
AMPK: AMP-activated protein kinase
Arg-1: arginase-1
ARS: Alizarin Red S
ATP: adenosine triphosphate
bFGF: basic fibroblast growth factor
BMP: bone morphogenetic protein
BV/TV: bone volume/total volume ratio
CCK-8: cell counting kit-8
cDNA: complementary DNA
CLSM: confocal laser scanning microscopy
CM: conditioned medium
COL-1: collagen type I
CS: citrate synthase
3D: three-dimensional
DAPI: 4',6-diamidino-2-phenylindole
DBM: decellularized bone matrix
dECM: decellularized extracellular matrix
DEG: differentially expressed gene
ECAR: extracellular acidification rate
ECM: extracellular matrix
EdU: 5-ethynyl-2'-deoxyuridine
ELISA: enzyme-linked immunosorbent assay
FTIR: Fourier transform infrared
G′: storage modulus
G″: loss modulus
GelMA: gelatin methacrylate
GPR: Gaussian Process Regression
GSEA: Gene Set Enrichment Analysis
GSH: reduced glutathione
GSSG: oxidized glutathione
H&E: hematoxylin and eosin
HUVEC: human umbilical vein endothelial cell
IDH1: isocitrate dehydrogenase 1
IL: interleukin
iNOS: inducible nitric oxide synthase
JBMSC: jaw bone marrow-derived mesenchymal stem cell
KEGG: Kyoto Encyclopedia of Genes and Genomes
LPS: lipopolysaccharide
micro-CT: micro-computed tomography
MSC: mesenchymal stem cell
NAD⁺: oxidized nicotinamide adenine dinucleotide
NADH: reduced nicotinamide adenine dinucleotide
OCN: osteocalcin
OCR: oxygen consumption rate
OPN: osteopontin
OXPHOS: oxidative phosphorylation
PBS: phosphate-buffered saline
PDLSC: periodontal ligament stem cell
PMA: phorbol 12-myristate 13-acetate
ROS: reactive oxygen species
RT-qPCR: real-time quantitative polymerase chain reaction
Runx2: Runt-related transcription factor 2
SD: standard deviation
SEM: scanning electron microscopy
sGAG: sulfated glycosaminoglycan
TCA: tricarboxylic acid
TEM: transmission electron microscopy
TGF-β1: transforming growth factor-beta1
TGF-β3: transforming growth factor-beta3
TNF-α: tumor necrosis factor-alpha
USC: urine-derived stem cell
USC-Exos: USC-derived exosomes
UV: ultraviolet
VEGF: vascular endothelial growth factor
VEGFA: vascular endothelial growth factor A
