# Supplementary material for: Flowerbed-inspired biomimetic 3D-printed scaffolds functionalized with urine-derived stem cell exosomes promote alveolar bone regeneration by regulating energy metabolism
Source: Theranostics. 2026 Jan 14;16(7):3826–56. doi: 10.7150/thno.123700 (PMC12846750; doi:10.7150/thno.123700)
Supplement: Supplementary file 1 — Supplementary methods and figures. [file thnov16p3826s1.pdf]

Supplementary material

Flowerbed-inspired biomimetic 3D-printed scaffolds functionalized with urine-derived stem cell exosomes promote alveolar bone regeneration by regulating energy metabolism

Supplementary experimental section

Cell isolation and culture

The human tissue acquisition and cell culture protocols used in this study were reviewed and approved by the Institutional Ethics Committee of the College of Stomatology, Chongqing Medical University (CQHS-REC-2024 [LSNo.119]). Written informed consent was obtained from all donors in accordance with established ethical guidelines. Urine-derived stem cells (USCs) were obtained from midstream morning urine samples collected from fifteen healthy male donors aged 20–30 years. Donors with a history of diabetes, autoimmune disorders, chronic infections, or other systemic diseases were excluded to ensure the quality and consistency of the cells. Each sample (50 mL) was centrifuged at 300 × *g* for 5 min at room temperature. The supernatant was then removed and the cell pellets were washed with phosphate-buffered saline (PBS) and resuspended in a specialized culture medium. The medium consisted of REBM® Basal Medium (Lonza, Switzerland) supplemented with REGM® SingleQuots® (Lonza, Switzerland), which supports the growth of renal epithelial cells. Fetal bovine serum (10%; FBS; Gibco, USA) and penicillin–streptomycin (1%) were also added to enhance cell proliferation and prevent microbial contamination. To minimize donor-to-donor variability and ensure consistent exosome quality, cells from primary USC cultures derived from all donors were pooled after expansion to passage 2 and used for subsequent exosome isolation. Jawbone marrow-derived mesenchymal stem cells (JBMSCs) were obtained from mandibular bone fragments discarded during orthognathic surgeries of systemically healthy donors aged 18–30 years with no history of periodontal disease,

systemic disorders, or medication use affecting bone metabolism. After thorough washing with PBS containing penicillin–streptomycin (1%) to remove blood residues, bone fragments were transferred to sterile flasks containing α-minimum essential medium (α-MEM; Gibco, USA) supplemented with FBS (10%) and penicillin–streptomycin (1%).

Flow cytometric analysis and multilineage differentiation capacity of USCs and JBMSCs

Flow cytometry was used to assess surface marker expression in passage-3 USCs and JBMSCs. Approximately 1×10^6 cells were suspended in PBS containing 2% FBS to ensure a single-cell suspension and incubated on ice for 30 min with FITC-labeled monoclonal antibodies against CD29, CD31, CD34, CD45, and CD90. Isotype-matched controls were used for gating. After staining, cells were washed twice in PBS under dark conditions to remove excess antibodies and analyzed using a BD Influx™ cytometer (BD Biosciences, USA).

To evaluate multipotency, osteogenic and adipogenic differentiation assays were performed on passage-3 cells. For osteogenesis, cells were plated in six-well plates at a density of 1×10^6 and grown to ~80% confluence before switching to osteoinductive medium. After 21 days, calcium mineralization was detected using Alizarin Red S staining (ARS; Beyotime, China). Adipogenic differentiation was initiated once cells reached full confluence, using a commercial adipogenic induction medium (Oricell, China). After 21 d of treatment, intracellular lipid droplets were visualized using Oil Red O staining (Solarbio, China) to identify their adipogenic potential.

Evaluation of the osteogenic inductive effect of USC-Exos on JBMSCs

The osteoinductive potential of exosomes derived from urine-derived stem cells

(USC-Exos) on JBMSCs was assessed using Cell Counting Kit-8 (CCK-8) assays, alkaline phosphatase (ALP) staining, and quantitative polymerase chain reaction (qPCR). For proliferation analysis, JBMSCs were seeded at 2×10^4 cells/well and treated with increasing concentrations of USC-Exos. Cell viability was determined on days 1, 3, and 5 using a CCK-8 kit (Elabscience, China) to evaluate dose-dependent effects on proliferation. To investigate early osteogenic differentiation, JBMSCs (1×10^6 cells/well) were cultured in osteogenic induction medium supplemented with varying concentrations of USC-Exos. After 7 days of induction, ALP staining was performed to assess early osteogenic activity. For gene expression analysis, total RNA was extracted on day 7, and qPCR was used to measure mRNA levels of osteogenic markers. Relative expression was calculated using the $2^{-}\Delta\Delta$Ct method.

Equilibrium swelling and degradation analysis

Hydrogel swelling properties were determined by initially lyophilizing and weighing hydrogel samples to establish their dry weight (Wd). These lyophilized samples were then immersed in PBS (3 mL; pH 7.4) at ambient temperature for 24 h to achieve equilibrium swelling. After swelling, samples were gently blotted dry and weighed (Ws). The swelling ratio was calculated as follows: Swelling ratio = (Ws − Wd)/Wd.

Hydrogel degradation was evaluated by first recording the initial dry weights (W_0) of samples. They were then incubated in a degradation solution containing collagenase types I and II (each at 1 μg/mL) at 37 °C with gentle agitation (120 rpm). At specific intervals, hydrogels were retrieved, washed, freeze-dried, and weighed again (W_1). The remaining mass percentage was computed as follows: Remaining mass (%) = $(W_1/W_0) \times 100\%$

Seahorse metabolic assay

Cellular metabolic activity was evaluated using a Seahorse XFe96 extracellular flux

analyzer (Agilent Technologies) by measuring the oxygen consumption rate (OCR) and extracellular acidification rate (ECAR). For mitochondrial respiration assays, Seahorse XF DMEM assay medium (Cat. No. 103576-100, Agilent) supplemented with 10 mM glucose, 1 mM pyruvate, and 2 mM L-glutamine was used. Following instrument calibration, mitochondrial stress testing was conducted using sequential injections of carbonyl cyanide-4-(trifluoromethoxy)phenylhydrazone (FCCP) and a mixture of rotenone and antimycin A (Rot/AA). To assess glycolytic function, cells were incubated in XF DMEM containing 2 mM L-glutamine. Glycolytic stress testing involved the sequential addition of glucose (10 mM), oligomycin (1 μM), and 2-deoxy-D-glucose (2-DG, 50 mM) to evaluate basal glycolysis, glycolytic capacity, and inhibition.

Quantitative real-time polymerase chain reaction

At the indicated time points, total RNA was extracted from the treated cells using an RNA Isolation Kit (Beyotime, China), and the RNA concentration was measured using a spectrophotometer (Thermo Scientific, USA). RNA (500 ng) was then converted into complementary DNA (cDNA) using a reverse transcription kit (Takara, Japan) in a 10 μL reaction volume. qPCR was performed with a SYBR-based kit (Takara, Japan) on a CFX Connect Real-Time PCR Detection System (Bio-Rad, USA). Relative gene expression was normalized to *β-actin* and calculated using the $2^{-\Delta\Delta CT}$ method. Primer sequences used for gene amplification are listed in Table S1 and were synthesized by Tsingke Biotech (China).

Enzyme-linked immunosorbent assay

Cell culture supernatants were collected after treatment, centrifuged (12,000 × g, 5 min) to remove debris, and analyzed using an enzyme-linked immunosorbent assay (ELISA). Inflammatory cytokines (interleukin [IL]-4, IL-10, IL-6, tumor necrosis factor-alpha [TNF-α], Elabscience, China) and regenerative markers (vascular

endothelial growth factor [VEGF], basic fibroblast growth factor [bFGF], bone morphogenetic protein [BMP]-2, transforming growth factor-beta1 [TGF-β1; Neobioscience, China]) were quantified. Assays were performed according to the manufacturer's instructions. Absorbance was measured using a microplate reader, and cytokine concentrations were derived from standard curves.

Western blot analysis

At each indicated time point, proteins were extracted using a radioimmunoprecipitation assay lysis buffer with phenylmethylsulfonyl fluoride (Beyotime, China). Protein quantification was achieved with a BCA protein assay kit (Beyotime, China). Equal protein amounts were separated using sodium dodecyl sulfate–polyacrylamide gel electrophoresis and transferred onto polyvinylidene fluoride membranes (Millipore, USA). The membranes were then blocked with 5% skim milk in Tris-buffered saline with Tween-20 and incubated overnight at 4 °C with the primary antibodies. They were then incubated with horseradish peroxidase-conjugated secondary antibodies (2 h, 37 °C). Bands were visualized using enhanced chemiluminescence reagent (Beyotime, China). Antibody details are provided in the Supplementary Material.

Immunofluorescence staining

At each indicated time point, cells were fixed (4% paraformaldehyde), permeabilized (0.2% Triton X-100), and blocked (5% bovine serum albumin). Primary antibody incubation occurred overnight at 4 °C, followed by incubation with fluorescent secondary antibodies (2 h, 37 °C). For mitochondrial staining, cells were labeled with MitoTracker Red CMXRos (Beyotime, China) for 10 min at 37 °C. Images were captured using a Leica fluorescence microscope. Antibody details are provided in the Supplementary Material.

In vitro immunomodulatory evaluation

To explore the immunomodulatory effects of hydrogel scaffolds on macrophage polarization, THP-1 monocytes were induced to differentiate into macrophage-like cells using 100 ng/mL phorbol 12-myristate 13-acetate (PMA; MCE, China) for 24–48 h. These macrophage-like cells (5×10^5 cells per well) were then cultured in six-well plates with various hydrogel scaffolds and stimulated with lipopolysaccharide (LPS; 1 μg/mL, Sigma, USA) to induce M1 polarization. Cells without hydrogels served as negative controls. Macrophage polarization and inflammatory responses were evaluated using immunofluorescence staining, Western blotting, quantitative PCR, and ELISA after co-culturing with a Transwell system (Corning, USA).

Conditioned media were harvested after 24 h of macrophage co-culture, centrifuged (5000 rpm, 5 min), and filtered through a 0.22 μm filter to eliminate cell debris. These conditioned media were mixed equally (1:1 v/v) with fresh α-MEM for JBMSCs or DMEM for human umbilical vein endothelial cells (HUVECs) cultures. Osteogenic potential was evaluated by culturing JBMSCs in conditioned medium supplemented with β-glycerophosphate disodium salt (10 mM), ascorbic acid (0.05 mM), and dexamethasone (10 nM), with the medium replaced every two days. ALP activity and ARS staining were performed to assess osteogenesis at specified intervals. Angiogenic potential was tested using HUVECs cultured in conditioned media through scratch wound healing and tube formation assays.

In vitro angiogenesis assessment

HUVECs were used to evaluate the angiogenic potential of hydrogel scaffolds *in vitro*, utilizing a co-culture system with a Transwell device (Corning, USA). Angiogenesis was comprehensively assessed using scratch wound migration, Transwell assays, tube formation, immunofluorescence staining, quantitative PCR, and ELISA. Tube formation involved seeding HUVECs (5×10^4 cells per well) onto Matrigel-coated 24-well plates (250 μL Matrigel per well, Corning, USA) followed

by incubation at 37 °C for 30 min before adding cells and scaffolds. Tubular structures were imaged after 6 h, and junction counts were analyzed using ImageJ software.

Hemolysis assay

Fresh whole blood was collected from rats and then centrifuged at 1500 rpm for 10 min to isolate red blood cells (RBCs). The RBCs were washed three times with PBS and resuspended in PBS to obtain a 2% (v/v) RBC suspension. Scaffold samples were incubated with the RBC suspension at 37 °C for 2 h. After centrifugation, the supernatant was collected and the absorbance at 540 nm was measured using a microplate reader. PBS and distilled water were used as the negative (0% hemolysis) and positive (100% hemolysis) controls, respectively.

Nanoindentation test

Scaffold mechanical properties were evaluated using a nanoindenter (Bruker, USA) equipped with a Berkovich diamond tip. Samples were fixed on a flat substrate, and indentations were performed using load-controlled mode: a maximum load of 2000 μN, a loading time of 10 s, a holding time of 10 s, and an unloading time of 10 s. Indentation depth was recorded continuously throughout the test. The elastic modulus and hardness were subsequently calculated.

Supplementary tables

Table S1. RT-qPCR primer sequences

Gene	Full name	Species	Primer	Sequences (5′–3′)
ALP	Alkaline phosphatase	Human	Forward	AACATCAGGGACATTGACGTG
			Reverse	GTATCTCGGTTTGAAGCTCTTCC
Runx2	Runt-related transcription factor 2	Human	Forward	CAGACCAGCAGCACTCCATA
			Reverse	TCATCTGGCTCAGATAGGAG
OSX	Osterix	Human	Forward	GAGGGCAGTAAGGTGGTGA
			Reverse	GCTGGGAAAGGAGGCATAA
bFGF	Basic fibroblast growth factor	Human	Forward	GGCTGTACTGCAAAAACGGG
			Reverse	CTGGTCCCGTTTTGGATCCG
Ang-1	Angiopoietin-1	Human	Forward	CAGGGAGACAGCAACAGCAA
			Reverse	GCTGGTGGTGTTGATGTTGA
HIF-1α	Hypoxia-inducible factor 1-alpha	Human	Forward	GAAAGCGCAAGTCTTCAAAG
			Reverse	TGGGTAGGAGATGGAGATGC
CD86	Cluster of differentiation 86	Human	Forward	CTGCACAGTGACCCTCACAT
			Reverse	CAGGAGGCAGGTGTTGTTGT
TNF-α	Tumor necrosis factor-alpha	Human	Forward	CCTCTCTCTAATCAGCCCTCTG
			Reverse	GAGGACCTGGGAGTAGATGAG
TGF-β	Transforming growth factor-beta	Human	Forward	GCCCTGGACACCAACTATTG
			Reverse	AGGTAACGCCAGGAATTGTT
CD206	Cluster of differentiation 206	Human	Forward	CTCTGTTCAGCTATTGGACGC
			Rever	CGGAATTTCTGGGATTCAGCT

			se	
CS	Citrate synthase	Human	Forward	GGCAGAGGTGACCAAGAAGA
			Reverse	CAGCCAGGTCCAGGTAGTTC
IDH1	Isocitrate dehydrogenase 1	Human	Forward	TGCCACCAACTGCAAGACCT
			Reverse	GCCACAGACACCATGACGAA
IDH2	Isocitrate dehydrogenase 2	Human	Forward	TGGGACCTGCAGAGAGATGA
			Reverse	GGCCTTGTTGATGTTGAGCA
OGDH	Oxoglutarate dehydrogenase	Human	Forward	GCTGGGACAGGTGTTTGTCA
			Reverse	CAGCCACAGGTGTTGGTAGA
NDUFB8	NADH: ubiquinone oxidoreductase subunit B8	Human	Forward	GCTGGTGGCTGTGTTTCTTC
			Reverse	CAGGCAGGTGTTGGTAGAGG
SDHB	Succinate dehydrogenase complex iron sulfur Subunit B	Human	Forward	GCCAGGTGACCTTCATCAAG
			Reverse	CAGGTTGGTGGTGTTGATGG
UQCRC2	ubiquinol-Cytochrome c reductase core protein 2	Human	Forward	GCTGGTGGCATTGTTCTCTG
			Reverse	CAGGCAGGTGTTGGTAGAGG
COX6B2	Cytochrome c oxidase subunit 6B2	Human	Forward	GCTGGTGGCTGTGTTTCTTC
			Reverse	CAGGCAGGTGTTGGTAGAGG
ATP5F1A	ATP synthase F1 subunit alpha	Human	Forward	GCCAGGTGACCTTCATCAAG
			Reverse	CAGGTTGGTGGTGTTGATGG
β-ACTIN	Actin beta	Human	Forward	CACCATTGGCAATGAGCGGTTC
			Reverse	AGGTCTTTGCGGATGTCCACGT

Table S2. Antibodies used in this study

Maker/specials	Category	Dilution	Category number/distributor/ source
**CD9 rabbit mAb**	Primary antibody	1:1000	ab236630, Abcam, USA
**CD63 rabbit mAb**	Primary antibody	1:1000	ab134045, Abcam, USA
**CD81 rabbit mAb**	Primary antibody	1:1000	ab109201, Abcam, USA
**Calnexin rabbit pAb**	Primary antibody	1:1000	ab22595, Abcam, USA
**CD86 rabbit mAb**	Primary antibody	1:5000	ET1606-50, HuaBio, China
**Arg-1 rabbit t pAb**	Primary antibody	1:5000	16001-1-AP, Proteintech, China
**ALP rabbit pAb**	Primary antibody	1:500	381009, ZenBio, China
**RUNX2 rabbit pAb**	Primary antibody	1:200	20700-1-AP, Proteintech, China
**OCN rabbit mAb**	Primary antibody	1:1000	A20800, Abclonal Technology, China
**NDUFB8 rabbit pAb**	Primary antibody	1:5000	14794-1-AP, Proteintech, China
**SDHB rabbit pAb**	Primary antibody	1:5000	10620-1-AP, Proteintech, China
**UQCRC2 rabbit pAb**	Primary antibody	1:2000	14742-1-AP, Proteintech, China
**MTCO2 rabbit pAb**	Primary antibody	1:2000	55070-1-AP, Proteintech, China
**ATP5F1 rabbit pAb**	Primary antibody	1:1000	15999-1-AP, Proteintech, China
**Phospho-AMPK rabbit mAb**	Primary antibody	1:1000	ab133448, Abcam, USA
**AMPK rabbit mAb**	Primary antibody	1:5000	ET1608-40, HuaBio, China
**iNOS rabbit pAb**	Primary antibody	1:200	18985-1-AP, Proteintech, China
**CD31 rabbit mAb**	Primary antibody	1:100	ab182981, Abcam, USA
**VEGFA rabbit rAb**	Primary antibody	1:100	81323-2-RR, Proteintech, China
**CS rabbit pAb**	Primary antibody	1:100	16131-1-AP, Proteintech, China

**IDH1 rabbit pAb**	Primary antibody	1:100	12332-1-AP, Proteintech, China
**CD206 rabbit pAb**	Primary antibody	1:100	18704-1-AP, Proteintech, China
**Col-1 rabbit mAb**	Primary antibody	1:100	67288-1-Ig, Proteintech, China
**CD90 rabbit mAb**	Primary antibody	1:50	ab307736, Abcam, USA
**F4/80 rabbit mAb**	Primary antibody	1:100	ab300421, Abcam, USA
**OXPHOS rabbit mAb**	Primary antibody	1:100	ab317271, Abcam, USA
**SOCS1 rabbit mAb**	Primary antibody	1:1000	HA723133, HuaBio, China
**STAT3 rabbit mAb**	Primary antibody	1:2000	ET1607-38, HuaBio, China
**Phospho-STAT3 (Y705) rabbit mAb**	Primary antibody	1:5000	ET1603-40, HuaBio, China
**NFκB p65 (F-6) mouse mAb**	Primary antibody	1:1000	SC-8008, Santa Cruz Biotechnology, USA
**p-NFκB p65 (27.Ser 536) mouse mAb**	Primary antibody	1:1000	SC-136548, Santa Cruz Biotechnology, USA
**Beta actin rabbit pAb**	Primary antibody	1:4000	20536-1-AP, Proteintech, China
**HRP-conjugated goat anti-rabbit IgG(H+L)**	Secondary antibody	1:10000	SA00001-2, Proteintech, China
**Goat anti-mouse IgG H&L (Alexa Fluor® 594)**	Secondary antibody	1:400	ab150116, Abcam, USA
**Goat anti-rabbit IgG H&L (Alexa Fluor® 488)**	Secondary antibody	1:400	ab150077, Abcam, USA

Supplementary Figures

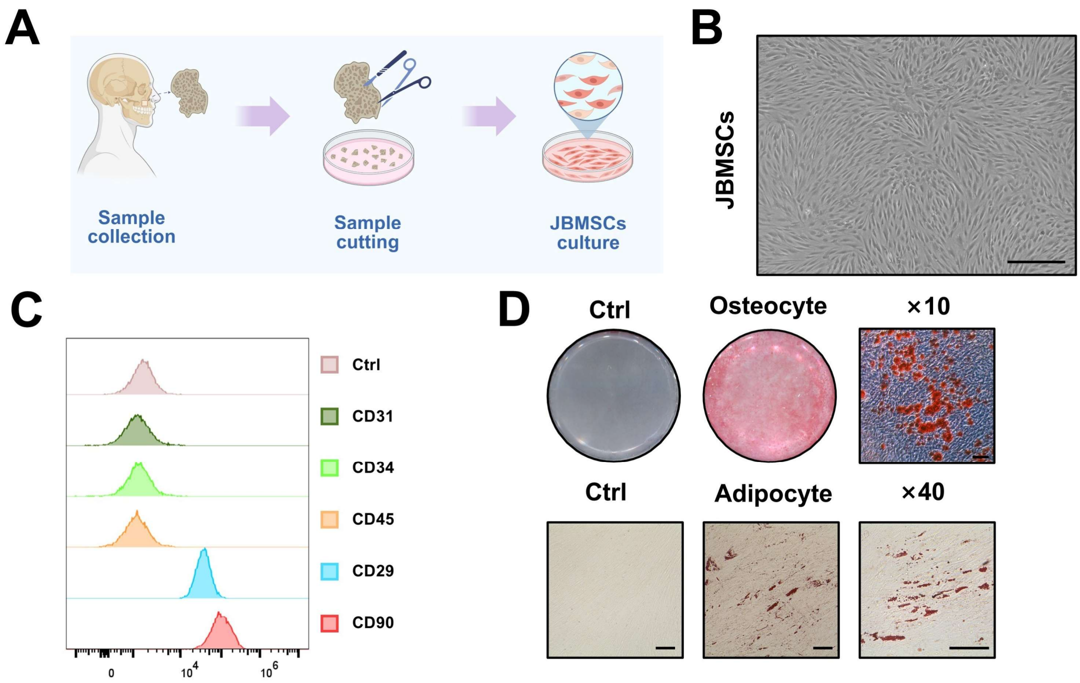

Figure S1. JBMSC isolation, characterization, and differentiation potential.

(A) Schematic workflow illustrating the collection, tissue cutting, and *in vitro* culture of JBMSCs. (B) Representative morphology of cultured JBMSCs under phase-contrast microscopy. Scale bar = 100 μm. (C) Flow cytometric analysis. (D) Multilineage differentiation of JBMSCs. Scale bars = 100 μm.

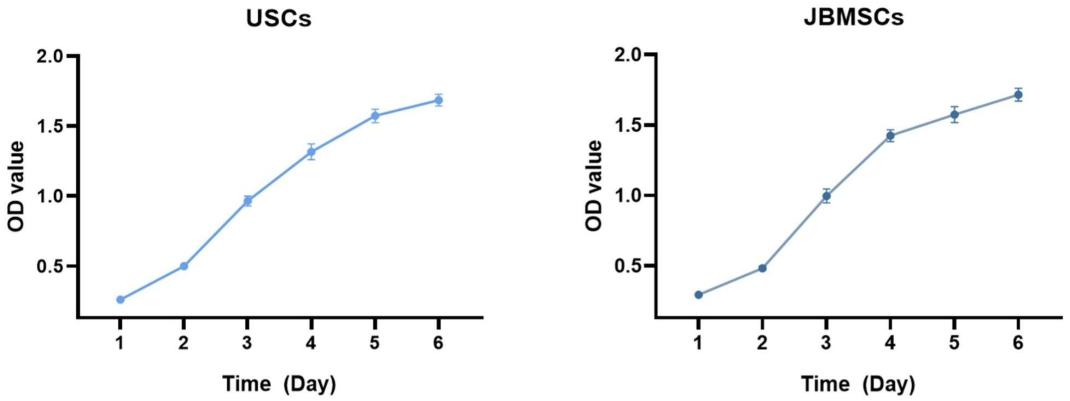

Figure S2. CCK-8 proliferation curves of JBMSCs and USCs over a 6-day culture period. All experiments were independently repeated at least three times. Data are presented as mean ± SD.

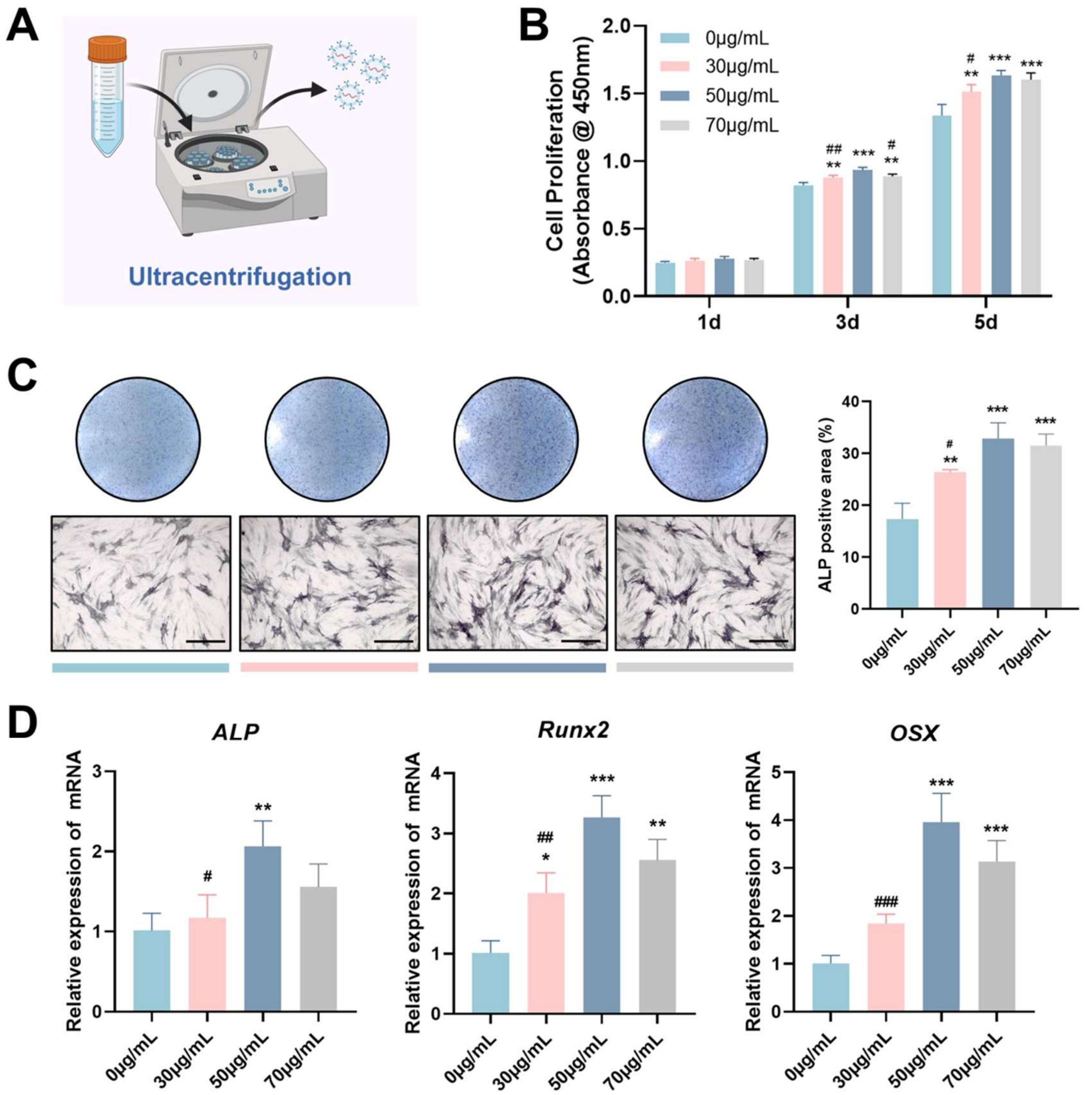

Figure S3. Exosome-mediated promotion of osteogenic differentiation in JBMSCs. (A) Schematic diagram of ultracentrifugation-based exosome isolation from USC culture medium. (B) CCK-8 assay showing dose-dependent proliferation of JBMSCs treated with varying concentrations of USC-Exos for 1, 3, and 5 days. (C) ALP staining and quantitative analysis of JBMSCs after 7 days of USC-Exos treatment at different doses. Scale bar = 400 μm. (D) RT-qPCR analysis of osteogenic gene expression levels in JBMSCs after USC-Exos treatment. All experiments were independently repeated at least three times. Data are presented as mean ± SD. * P < 0.05, ** P < 0.01, and *** P < 0.001 versus the 0 μg/mL group and $^{\#}$ P < 0.05, $^{\#\#}$ P < 0.01, and $^{\#\#\#}$ P < 0.001 versus the 50 μg/mL group.

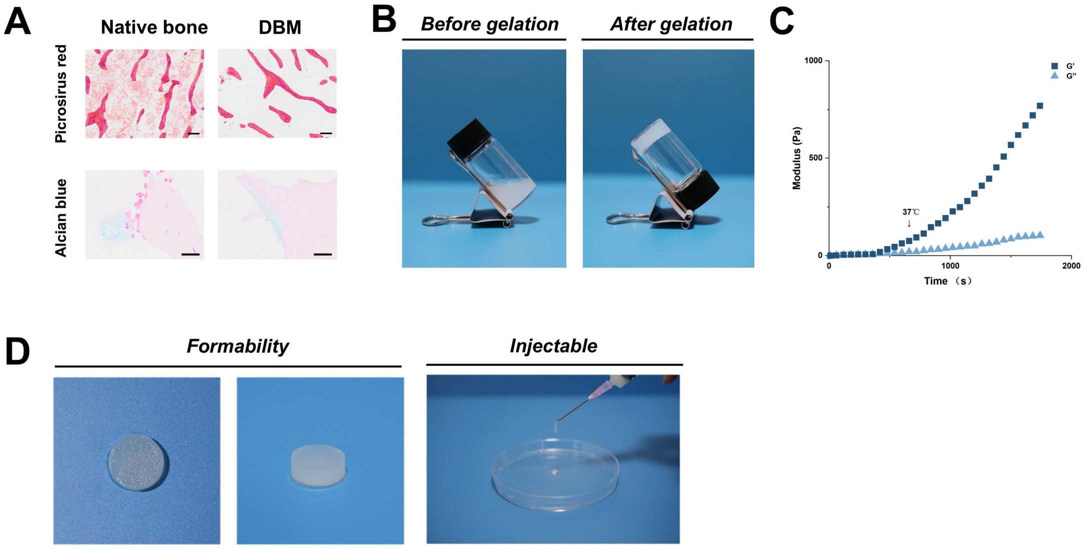

Figure S4. DBM preparation and evaluation. (A) Picrosirius Red (scale bar = 200 μm) and Alcian Blue (scale bar = 25 μm) staining of bone tissue before and after decellularization. (B) Photographs showing the pre-gel solutions of DBM before and after gelation at 37 °C. (C) Rheological analysis demonstrating the thermosensitive sol–gel transition behavior of the DBM pre-gel solution. (D) Formability and injectability of GelMA/DBM hydrogels.

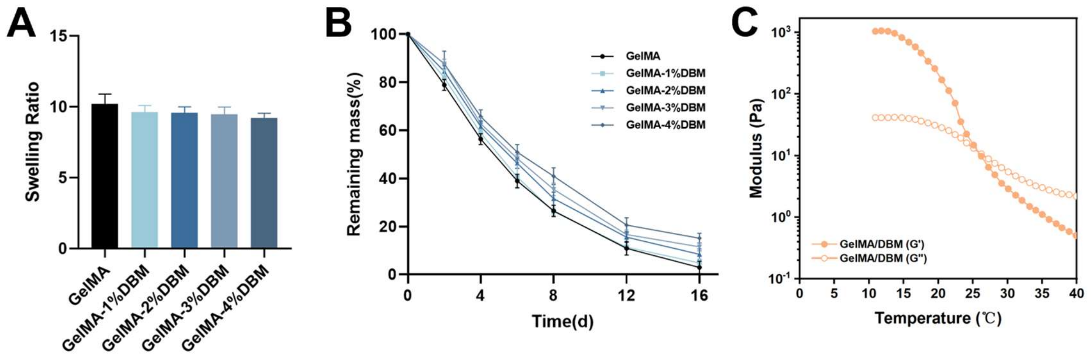

Figure S5. Swelling behavior, enzymatic degradation, and rheological properties of GelMA/DBM hydrogels. (A) Swelling ratio of GelMA hydrogels with increasing concentrations of DBM. (B) *In vitro* degradation profiles of GelMA hydrogels incorporating varying DBM contents. (C) Temperature-dependent rheological analysis of GelMA/DBM bioink. All experiments were independently repeated at least three times. Data are presented as mean ± SD.

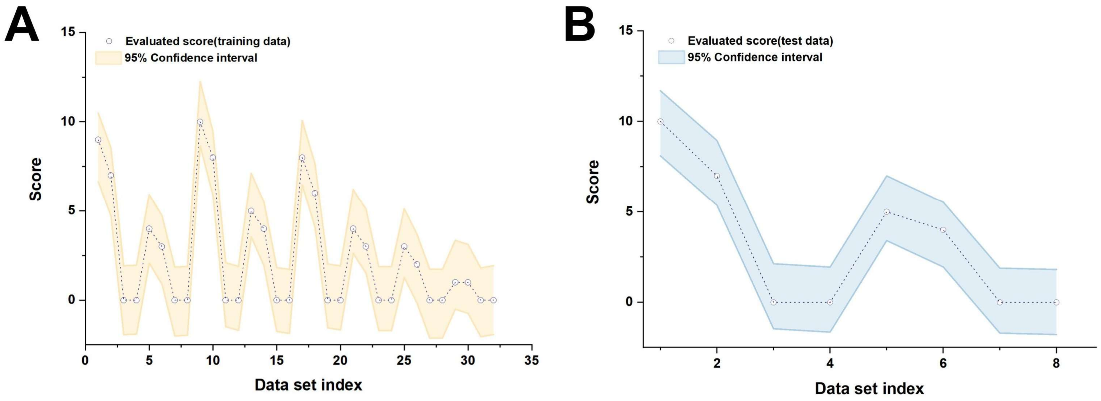

Figure S6. Evaluation of the machine learning model with a 95% confidence interval when using the training (A) and test (B) datasets.

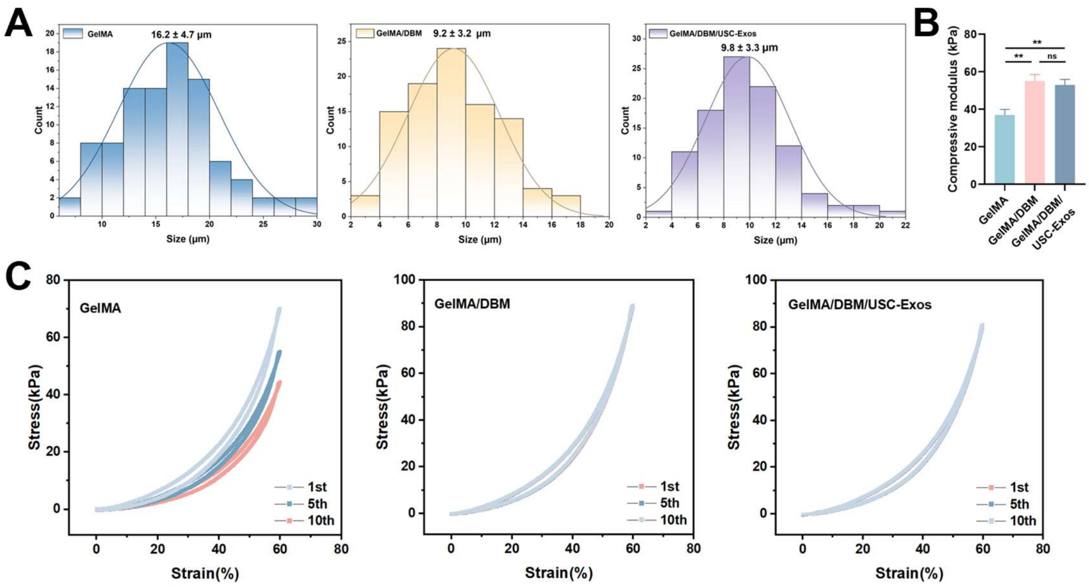

Figure S7. Characterization of hydrogel scaffolds. (A) Pore size distribution of GelMA, GelMA/DBM, and GelMA/DBM/USC-Exos scaffolds. (B) Compressive modulus for three types of hydrogel scaffolds. (C) Compressive stress–strain curves of different scaffolds under cyclic loading. All experiments were independently repeated at least three times. Data are presented as mean ± SD. * $P < 0.05$, ** $P < 0.01$, *** $P < 0.001$ versus the GelMA group.

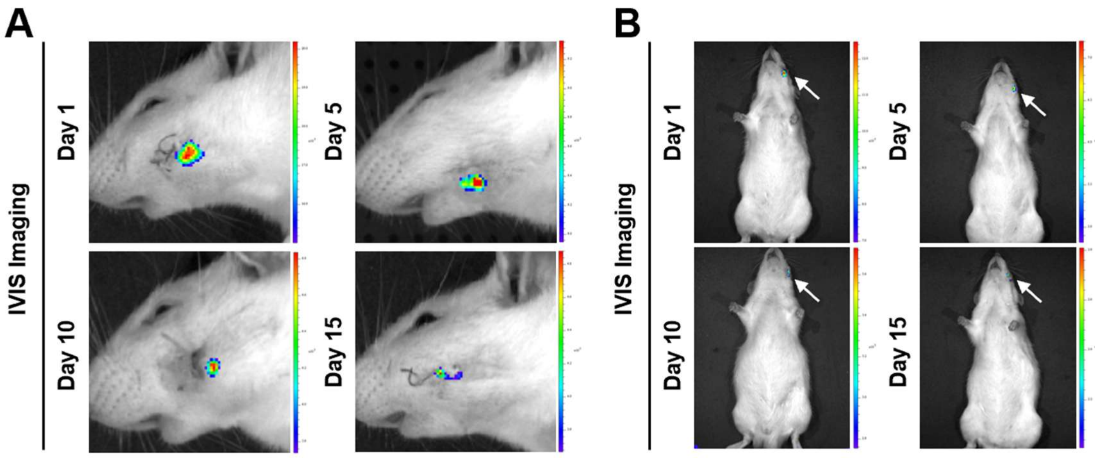

Figure S8. Local retention of the GelMA/DBM/USC-Exos hydrogel observed using an *in vivo* imaging system (IVIS). (A) Representative side-view IVIS images of the defect sites. (B) Representative whole-body IVIS images.

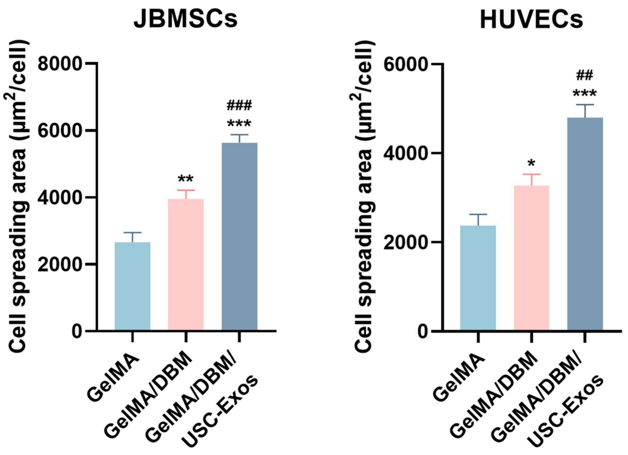

Figure S9. Quantitative analysis of the cell spreading area of JBMSCs and HUVECs cultured on different hydrogel scaffolds. All experiments were independently repeated at least three times. Data are presented as mean ± SD. * P < 0.05, ** P < 0.01, and *** P < 0.001 versus the GelMA group; $^{\#}$ P < 0.05, $^{\#\#}$ P < 0.01, and $^{\#\#\#}$ P < 0.001 versus the GelMA/DBM group.

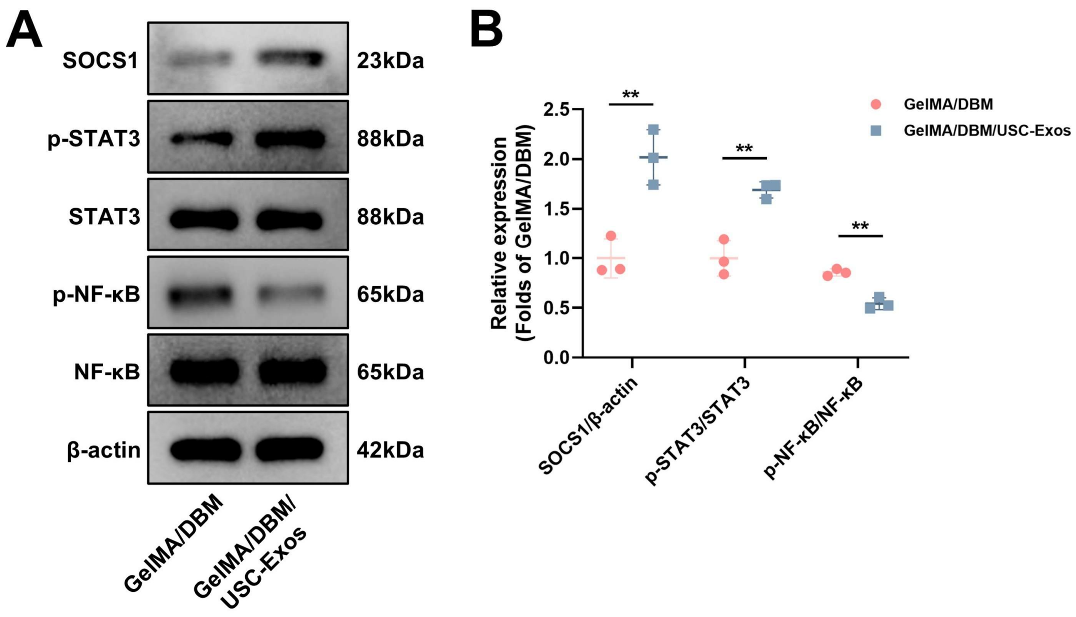

Figure S10. GelMA/DBM/USC-Exos scaffolds regulate macrophage polarization–associated signaling pathways. (A) Western blot analysis of key proteins involved in the SOCS1/STAT3 and NF-κB signaling pathways in macrophages cultured with GelMA/DBM or GelMA/DBM/USC-Exos scaffolds. (B) Quantitative analysis of SOCS1/β-actin, p-STAT3/STAT3, and p-NF-κB/NF-κB ratios, normalized to the GelMA/DBM group. Data are presented as mean ± SD ($n \geq 3$). * $P < 0.05$, ** $P < 0.01$, and *** $P < 0.001$ compared with the GelMA/DBM group.

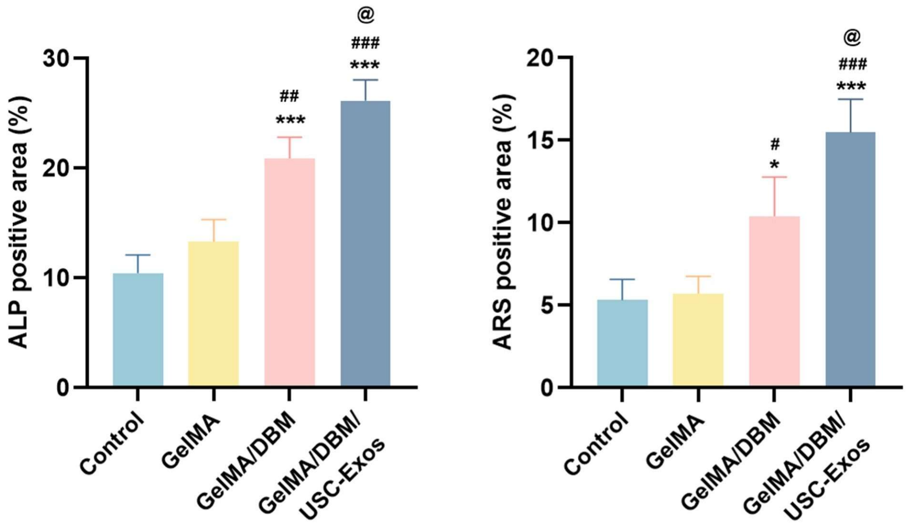

Figure S11. Quantitative analysis of ALP and ARS staining in different hydrogel groups. All experiments were independently repeated at least three times. Data are presented as mean ± SD. * P < 0.05, ** P < 0.01, and *** P < 0.001 versus the control group; $^{\#}$ P < 0.05, $^{\#\#}$ P < 0.01, and $^{\#\#\#}$ P < 0.001 versus the GelMA group; and $^{@}$ P < 0.05, $^{@@}$ P < 0.01, and $^{@@@}$ P < 0.001 versus the GelMA/DBM group.

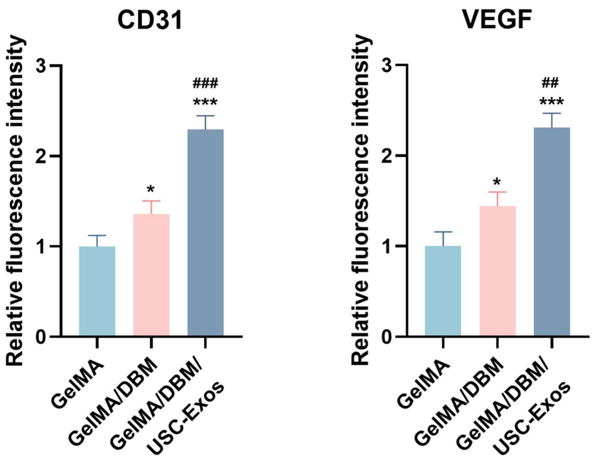

Figure S12. Quantitative fluorescence analysis of CD31 and VEGFA expression in HUVECs cultured on different hydrogel scaffolds. All experiments were independently repeated at least three times. Data are presented as mean ± SD. * P < 0.05, ** P < 0.01, and *** P < 0.001 versus the GelMA group; # P < 0.05, ## P < 0.01, and ### P < 0.001 versus the GelMA/DBM group.

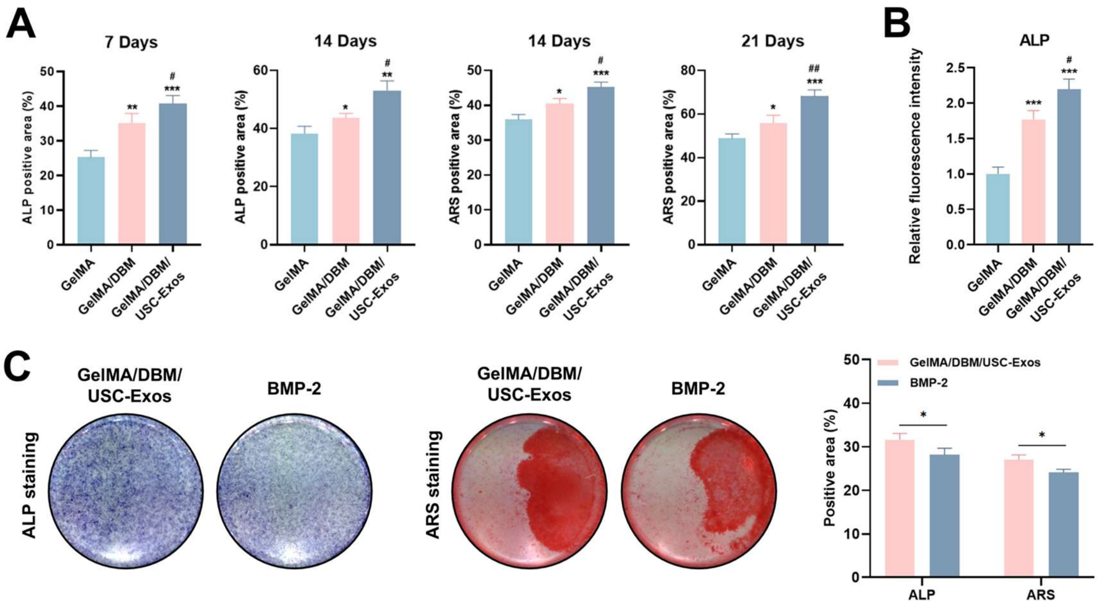

Figure S13. Osteogenic differentiation in different hydrogel groups. (A) Quantification of ALP- and ARS-positive areas. (B) Quantitative fluorescence analysis of ALP expression in different hydrogel groups. (C) ALP and ARS staining of JBMSCs under BMP-2 and GelMA/DBM/USC-Exos conditions. All experiments were independently repeated at least three times. Data are presented as mean ± SD. * $P < 0.05$, ** $P < 0.01$, and *** $P < 0.001$ versus the GelMA group; $^{\#}$ $P < 0.05$, $^{\#\#}$ $P < 0.01$, and $^{\#\#\#}$ $P < 0.001$ versus the GelMA/DBM group.

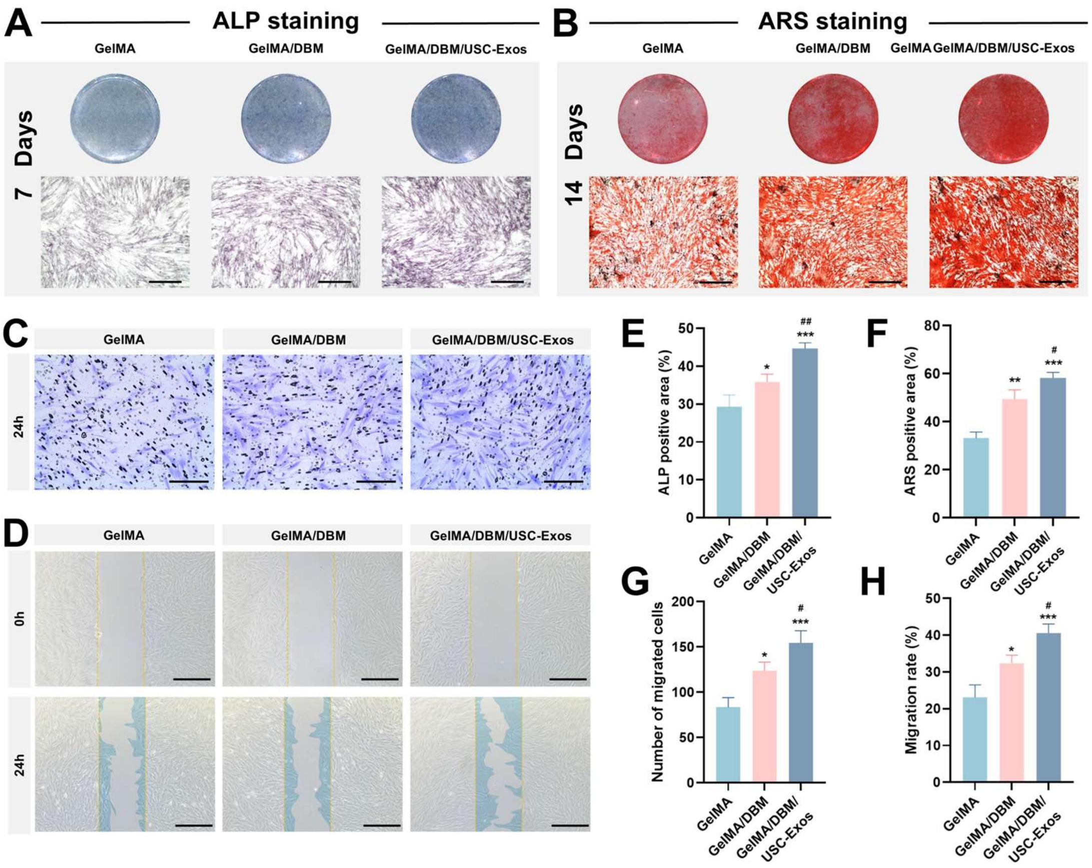

Figure S14. Osteogenic and recruitment responses of PDLSCs to GelMA/DBM/USC-Exos scaffolds. (A) ALP staining of PDLSCs after 7 days of osteogenic induction. Scale bar = 400 μm. (B) ARS staining of PDLSCs after 14 days to assess mineralized matrix formation. Scale bar = 400 μm. (C) Transwell migration assay of PDLSCs after 24 h. Scale bar = 200 μm. (D) Scratch assay showing wound closure dynamics over 24 h. Scale bar = 400 μm. (E–F) Quantitative analysis of ALP- and ARS-positive areas. (G–H) Number of migrated cells and migration rate. All experiments were independently repeated at least three times. Data are presented as mean ± SD. * $P < 0.05$, ** $P < 0.01$, and *** $P < 0.001$ versus the GelMA group; $^{\#}$ $P < 0.05$, $^{\#\#}$ $P < 0.01$, and $^{\#\#\#}$ $P < 0.001$ versus the GelMA/DBM group.

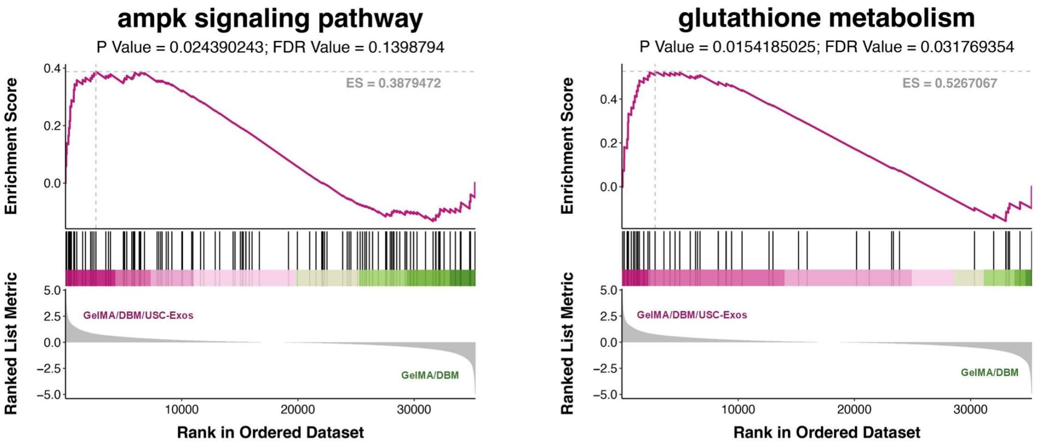

Figure S15. Gene set enrichment analysis (GSEA) of the AMPK signaling pathway and glutathione metabolism.

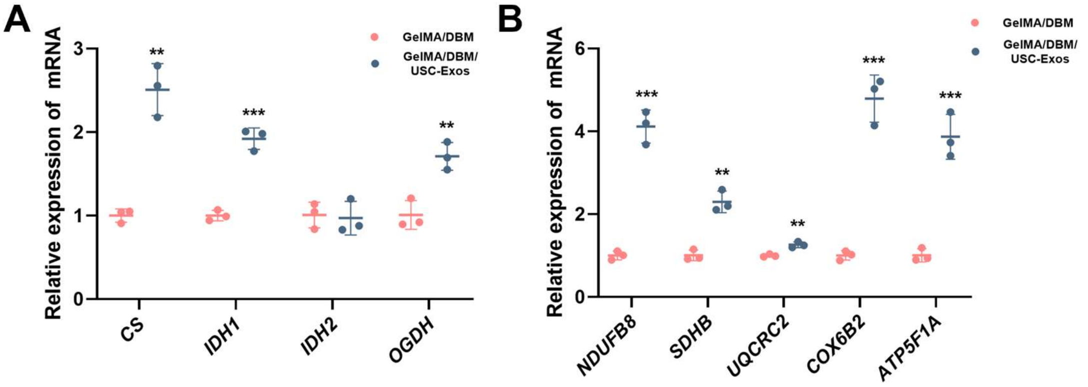

Figure S16. qPCR analysis of key metabolic genes involved in (A) the TCA cycle and (B) oxidative phosphorylation pathways. All experiments were independently repeated at least three times. Data are presented as mean ± SD. * $P < 0.05$, ** $P < 0.01$, and *** $P < 0.001$ versus the GelMA/DBM group.

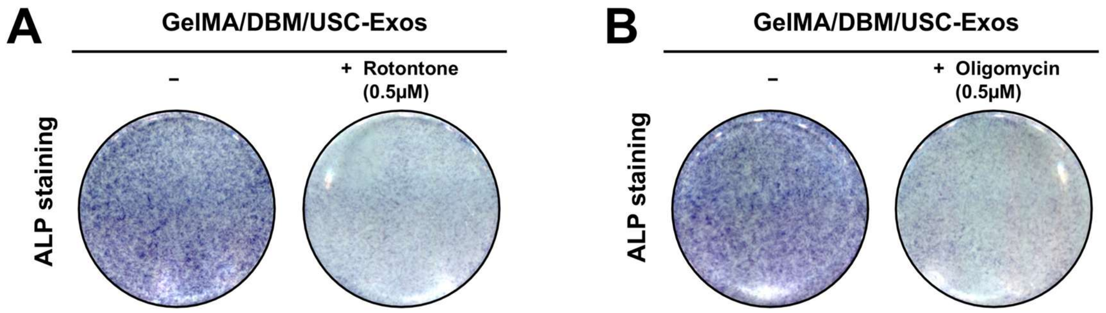

Figure S17. ALP staining of JBMSCs cultured with GelMA/DBM/USC-Exos scaffolds in the presence of metabolic inhibitors. (A) Rotenone (0.5 μM) inhibition. (B) Oligomycin (0.5 μM) inhibition.

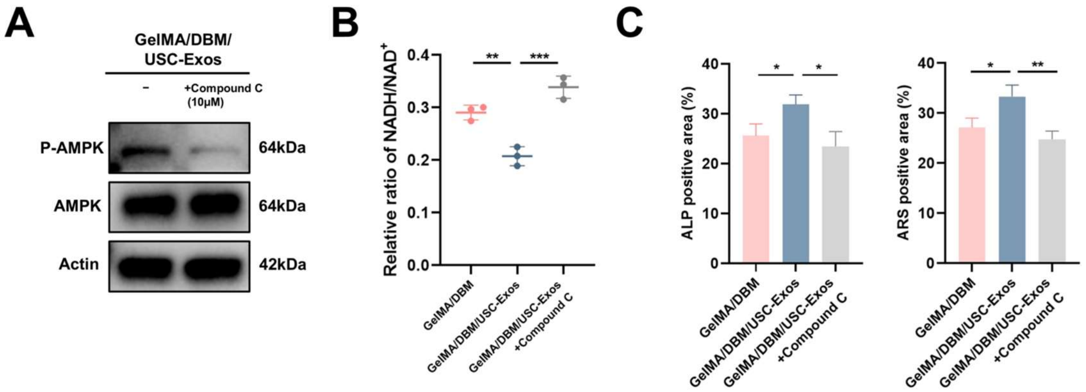

Figure S18. Validation of AMPK inhibition effects on mitochondrial function and osteogenesis in JBMSCs. (A) Western blot analysis showing that AMPK inhibitor Compound C significantly suppressed AMPK phosphorylation. (B) Quantification of the intracellular NADH/NAD^+ ratio in JBMSCs treated with GelMA/DBM/USC-Exos, with or without Compound C. (C) Quantitative analysis of ALP-positive and ARS-positive areas in JBMSCs following treatment with GelMA/DBM/USC-Exos, with or without Compound C. All experiments were independently repeated at least three times. Data are presented as mean ± SD. * P < 0.05, ** P < 0.01, and *** P < 0.001 versus the GelMA/DBM group.

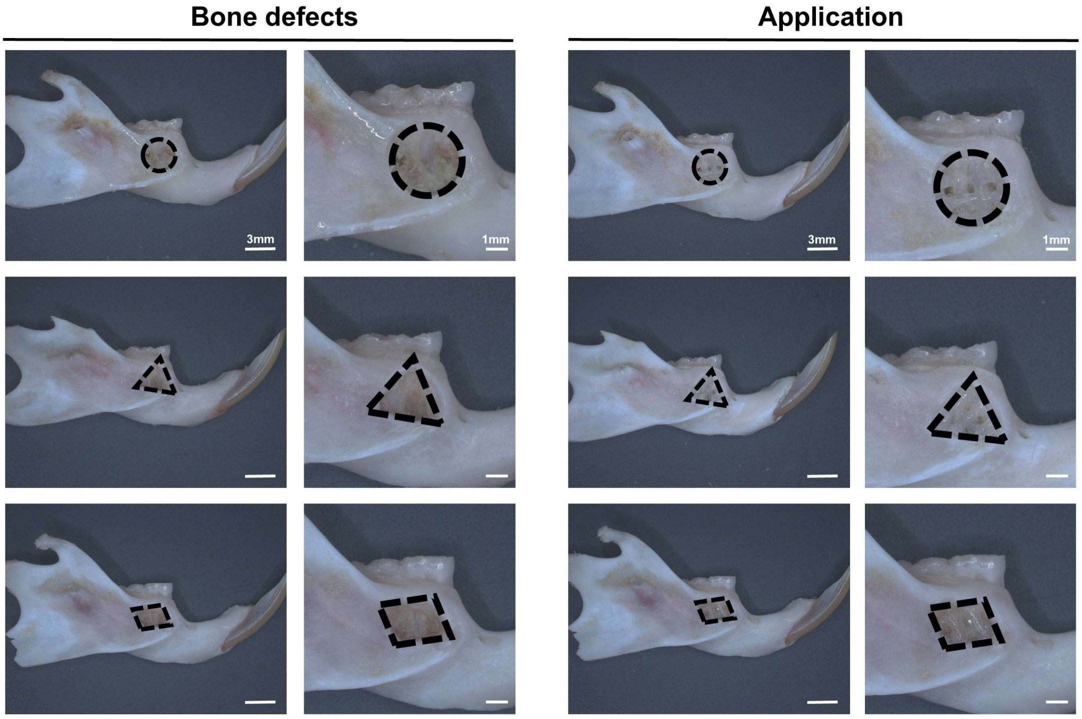

Figure S19. Representative photographs of *in vivo* mandibular alveolar bone defects with various geometric shapes established in rat models.

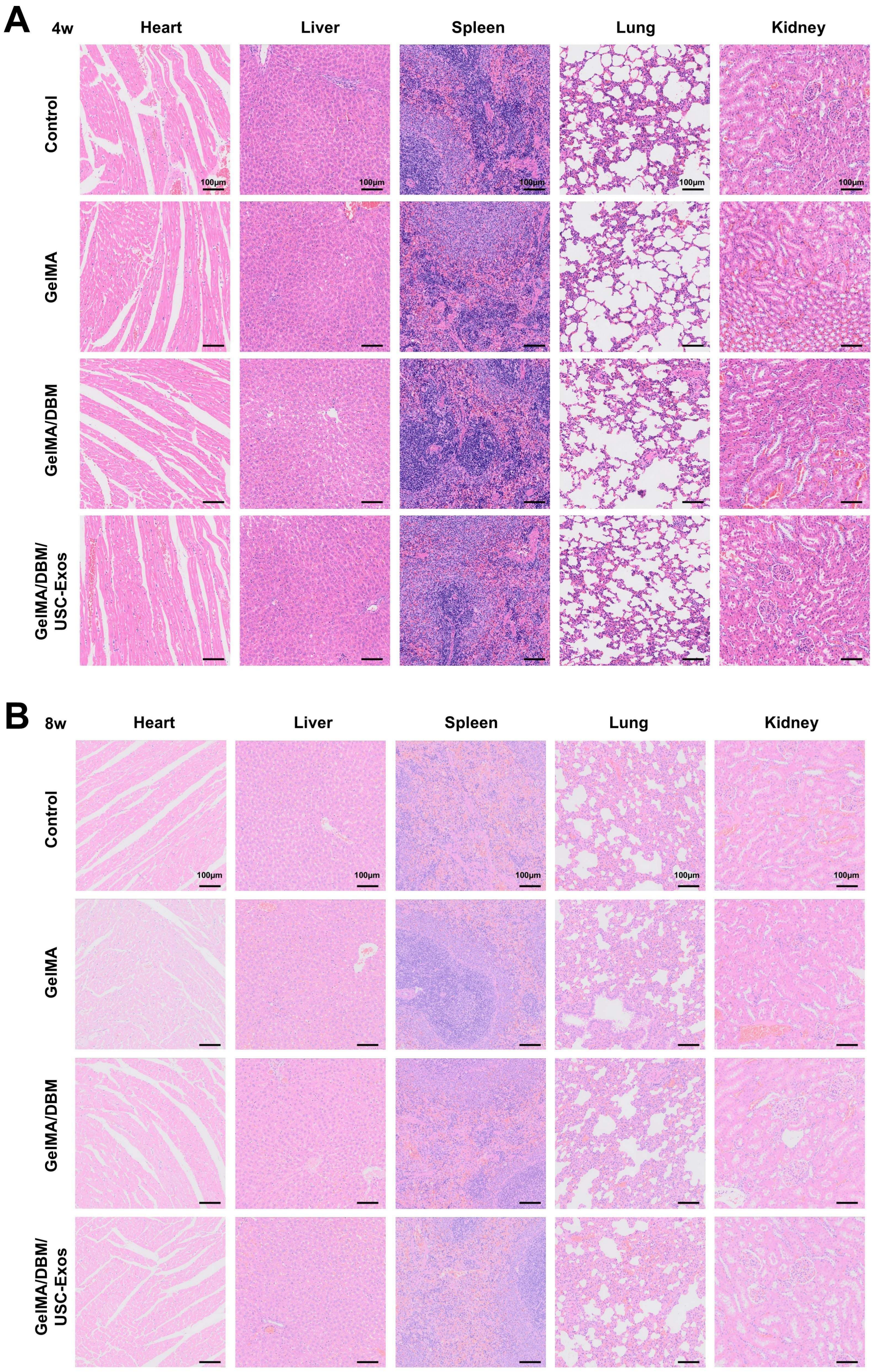

Figure S20. H&E staining of heart, liver, spleen, lung, and kidney samples from Sprague-Dawley rats 4 (A) and 8 (B) weeks after they were implanted with hydrogel scaffolds. No apparent histopathological abnormalities were observed. Scale bar = 100 μm.

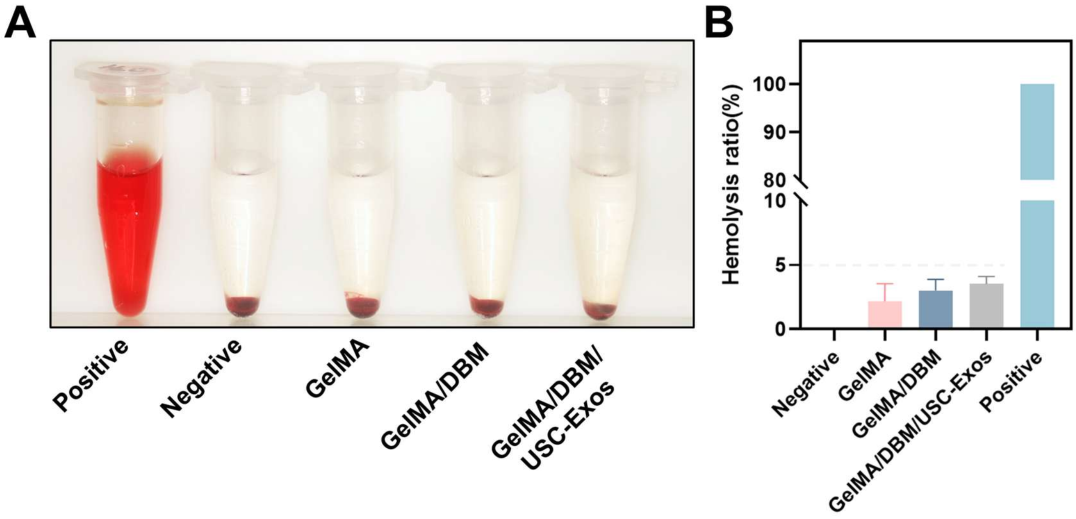

Figure S21. Scaffold hemolysis assay. (A) Hemolysis images of red blood cells incubated with different hydrogels. (B) Quantitative analysis of the hemolysis ratio (%).

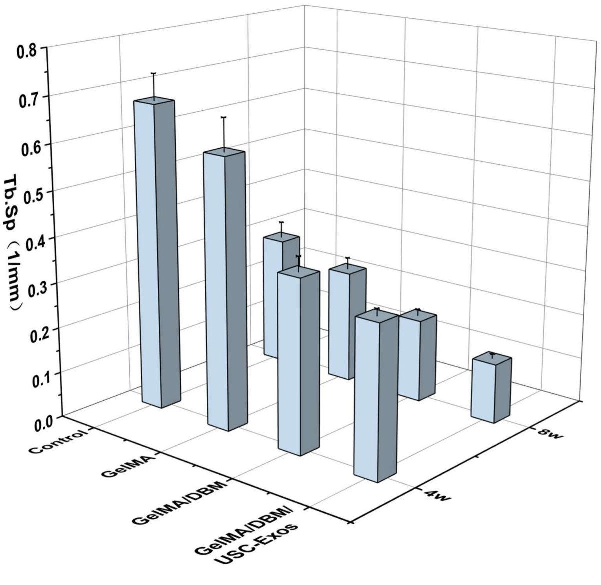

Figure S22. Quantitative analysis of Tb.Sp. All experiments were independently repeated at least three times. Data are presented as mean ± SD.

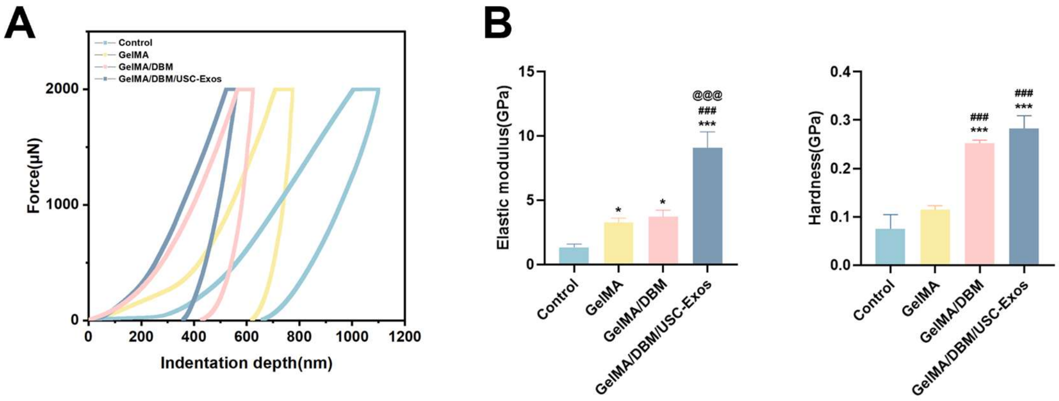

Figure S23. Nanoindentation analysis of regenerated bone tissue. (A) Representative load–displacement curves obtained from nanoindentation tests. (B) Quantitative comparison of the elastic modulus and hardness of newly formed bone within the defect regions. All experiments were independently repeated at least three times. Data are presented as mean ± SD. * $P < 0.05$, ** $P < 0.01$, and *** $P < 0.001$ versus the control group; # $P < 0.05$, ## $P < 0.01$, and ### $P < 0.001$ versus the GelMA group; and @ $P < 0.05$, @@ $P < 0.01$, and @@@ $P < 0.001$ versus the GelMA/DBM group.

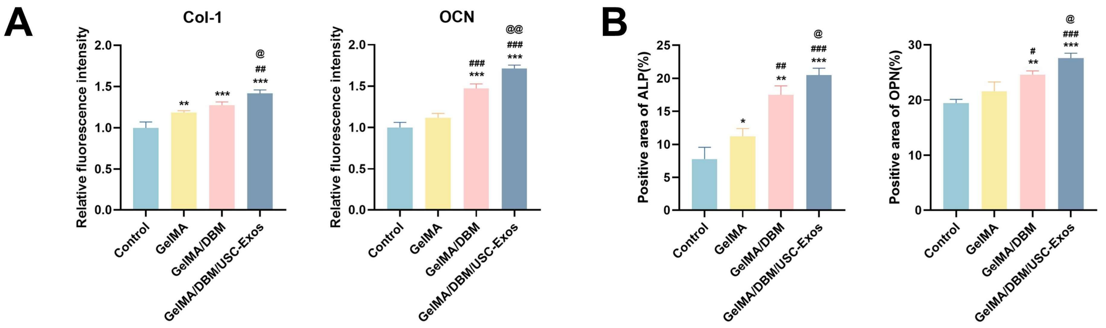

Figure S24. Quantitative analysis of osteogenic protein expression. (A) Quantification of Col-1 and OCN expression levels. (B) Quantification of ALP and OPN expression levels. All experiments were independently repeated at least three times. Data are presented as mean ± SD. * P < 0.05, ** P < 0.01, and *** P < 0.001 versus the control group; $^{\#}$ P < 0.05, $^{\#\#}$ P < 0.01, and $^{\#\#\#}$ P < 0.001 versus the GelMA group; and $^{@}$ P < 0.05, $^{@@}$ P < 0.01, and $^{@@@}$ P < 0.001 versus the GelMA/DBM group.

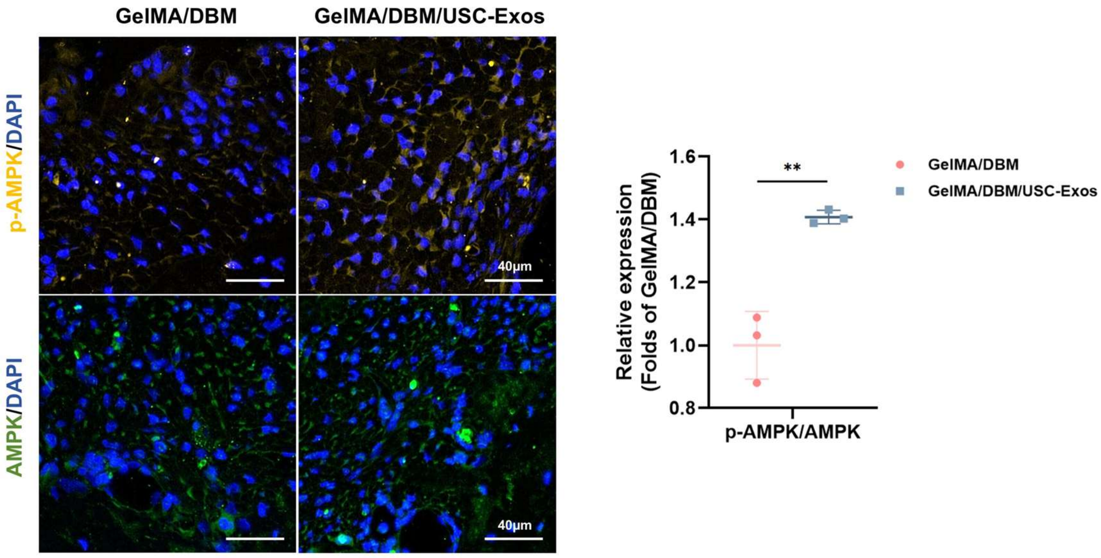

Figure S25. Immunofluorescence analysis of AMPK activation in regenerated bone tissue. All experiments were independently repeated at least three times. Data are presented as mean ± SD. * $P < 0.05$, ** $P < 0.01$, and *** $P < 0.001$ versus the GelMA/DBM group.
